# Entrepreneurial Leadership in Business Organizations: a systematic review and meta-analysis

**DOI:** 10.12688/f1000research.175169.1

**Published:** 2026-03-05

**Authors:** Agustin Amborowati, Asri Laksmi Riani, Mugi Harsono, Joko Suyono

**Affiliations:** 1 Departement of Management, Faculty Economic and Business, Universitas Sebelas Maret, Surakarta, Central Java, 57126, Indonesia; 2 Departement of Business Management, Vocational School, Universitas Sebelas Maret, Surakarta, Central Java, 57126, Indonesia

**Keywords:** Entrepreneurial Leadership, Leadership, Leadership Styles, Leadership in Entrepreneurship, Systematic Literature Review

## Abstract

This systematic literature review provides a comprehensive analysis of the current state of research on entrepreneurial leadership over the past decade. The study addresses key research questions, including publication frequency, productive countries, organizations, and journals, most researched variables, and most cited theories and influential authors. PRISMA flowchart was used, comprising four main stages: identification, screening, eligibility, and inclusion. Initially, 364 articles were identified through database searches; at the eligibility stage, 83 articles underwent full-text review. Findings reveal a significant increase in publications on entrepreneurial leadership, with Indonesia emerging as the most productive country and the Journal of Small Business Management as the most cited journal. Key variables such as employee creativity, entrepreneurial leadership, project success, and creative self-efficacy are frequently studied, with Social Cognitive Theory and Social Learning Theory being prominent theoretical foundations. While the study offers valuable insights, it acknowledges certain limitations, including potential publication bias due to the reliance on specific databases and the underrepresentation of contributions from developing countries. This review not only identifies key themes and trends but also proposes a conceptual framework for further research and practical insights for business leaders and entrepreneurs.

## Introduction

Awad et al. (2024) conducted a cross-sectional study among undergraduate nursing students in Thailand, revealing that a significant number of students exhibit high levels of entrepreneurial leadership. The entrepreneurial characteristics of nursing education can improve students' skills and readiness for innovative healthcare practices. Clark et al., (2023) highlighting the implementation of entrepreneurial leadership (EL) in the public sector, EL plays a role in promoting the principles of democratic principles and can improve organizational effectiveness. Soomro et al., (2019) Observing entrepreneurial leadership behaviors among college students, they showed that activities that promote cognitive flexibility can significantly improve students' entrepreneurial leadership behaviors, thus preparing them for future leadership roles in diverse organizational settings.

An essential point in entrepreneurial leadership is to consider the concept of open innovation ( Ataei et al., 2024a). Research related to EL has been widely conducted, some focusing on individual units of analysis ( Abualoush et al., 2022; Cai et al., 2019; K. Ercantan et al., 2024; Hoang, Luu, et al., 2023a; Hou et al., 2024c; Iqbal et al., 2022; Islam & Asad, 2024; Yang & Bentein, 2023) and organizations ( Khan et al., 2024; Paudel, 2019; Sarabi et al., 2020; Sarmawa et al., 2020; Sultan et al., 2023; Wu et al., 2021). This systematic literature review aims to explore research related to EL that has been carried out so far. This study synthesizes documents based on a systematic review in four stages, similar to the approach used by ( Hosany et al., 2022). However, it was further developed. The study is also expected to (1) identify key themes and trends in entrepreneurial leadership research, (2) develop a conceptual framework that can be used for further research, (3) provide practical insights for business leaders and entrepreneurs on how to apply entrepreneurial leadership principles to achieve success and innovation.

## Literature review

The focus on the examination of entrepreneurial leadership can traditionally be divided into two, depending on whether it is initially viewed from an entrepreneurial or leadership perspective ( Leitch & Volery, 2017). Integrating the latest technology, innovation, resources, opportunities, and added value will determine the success of a business ( Chaniago, 2023). The complexity of the relationship between organizational culture and entrepreneurial leadership and the application of a combined model of reflective practitioners and learner organizations in innovative leadership ( Shiferaw et al., 2023).

The conceptualization of EL can determine the direction of an organization and have a dynamic process ( Harrison et al., 2016). Although attention is focused, entrepreneurship and leadership are still ambiguous concepts ( Harrison et al., 2016). Shiferaw et al., (2023) conducting a systematic literature review, revealing the intricate relationships between entrepreneurial leadership, organizational learning, and organizational culture. Entrepreneurial leadership skills are still broad and largely unstructured ( Harrison et al., 2016). Norena-Chavez & Thalassinos, (2023) entrepreneurial leadership, not only at the leadership level but also throughout the organizational hierarchy. Djalil et al. (2023) demonstrating the important role of entrepreneurial leadership in improving the bank's performance through market orientation and innovation. The field of leadership and entrepreneurship provides essential insights into how individuals and organizations function and work in complex environments ( Harrison et al., 2016). Hensellek et al., (2023) entrepreneurial leadership affects performance in various kinds of organizations, such as new ventures, small- and medium-sized enterprises, and established firms.

Entrepreneurial Leadership (EL) is a leadership style that focuses on recognition, exploitation of opportunities, and empowering teams to create value in organizations. Gupta et al., (2004), added that EL includes the scenario enactment dimensions (creating a vision and mitigating uncertainty) and cast enactment (building team commitment). In an empirical context, Latif et al., (2020) shows that EL has a significant impact on the success of the project through the mediating role of the knowledge management process, such as the acquisition and utilization of strategic information. ( Mehmood, Jian, & Akram, 2020a) found that EL enhances employee creativity through knowledge sharing, with the added influence of learning orientation.

EL is also relevant in cross-cultural contexts. ( Gupta et al., 2004) identified that EL is globally recognized as a characteristic of effective leadership, although there are variations in its implementation depending on organizational culture and power hierarchies. ( Renko et al., 2015) developed the ENTRELEAD scale to quantitatively measure EL attributes, with high validity in various organizational contexts. EL requires determination to create solutions to various challenges, reducing uncertainty and risk at various stages of business development ( Hussain & Li, 2022). This leadership style is not limited to the organizational level, but rather focuses on exploiting opportunities and developing value through route clearing to achieve goals ( Hussain & Li, 2022). Although individual attention is an important component of transformational leadership, it is not a key element in EL ( Hussain & Li, 2022).

According to ( Hoang et al., 2022), EL plays an important role in improving the company's performance, while Taleb et al., (2023) emphasizes that EL helps micro, small, and medium enterprises (MSMEs) to survive and achieve success. Therefore, governments and socio-economic development institutions should focus more on developing entrepreneurial leadership skills, especially among low-income entrepreneurs ( Taleb et al., 2023). Gupta et al., (2004) explains that EL is a different type of leadership from traditional leadership styles, as it prioritizes resource mobilization and a commitment to creating value. These two challenges are interrelated, because the vision will be meaningless without supporters who are able to carry it out ( Gupta et al., 2004). Through cross-cultural research using data from Global Leadership and Organizational Behavior Effectiveness (GLOBE), EL is recognized as a universal leadership style that is relevant in various societies and is able to adapt to different social contexts ( Gupta et al., 2004).

## Methods

This literature review aims to synthesize and critically analyze existing research on the interaction between entrepreneurial leadership and organizational outcomes, focusing on research variables, hypotheses, and theoretical foundations. It offers a comprehensive overview of how different studies conceptualize and measure key variables such as innovation performance, organizational culture, and entrepreneurial success. This research employs a systematic approach, beginning with a literature search and thematic analysis. The article is structured as follows: first, defining and discussing the core characteristics of entrepreneurial leadership; second, examining various theoretical frameworks in the literature; third, reviewing common variables in the literature; and finally, analyzing prevalent themes, identifying gaps and challenges, and proposing implications for future research and practice.

Ideally, all papers on entrepreneurial leadership would be included in this review ( Helden et al., 2023). Alternatively, a representative sample of papers relevant to conceptual development in entrepreneurial leadership could be included. However, due to the extensive research in this field and the numerous articles identified through our search and screening process, including all relevant articles would be impractical and time-consuming. Therefore, a feasible alternative is employing theoretical sampling as per the PRISMA guidelines to achieve the study's objectives. Initially, articles were selected from a population of 364 articles from Scopus. To illustrate the article selection process, a PRISMA flowchart ( Helden et al., 2023) was used, comprising four main stages: identification, screening, eligibility, and inclusion. Initially, 364 articles were identified through database searches. After excluding ineligible articles (titles, abstracts, and irrelevance), 83 articles underwent full-text review, with all 83 ultimately included in the final systematic review.

**Figure 1.**
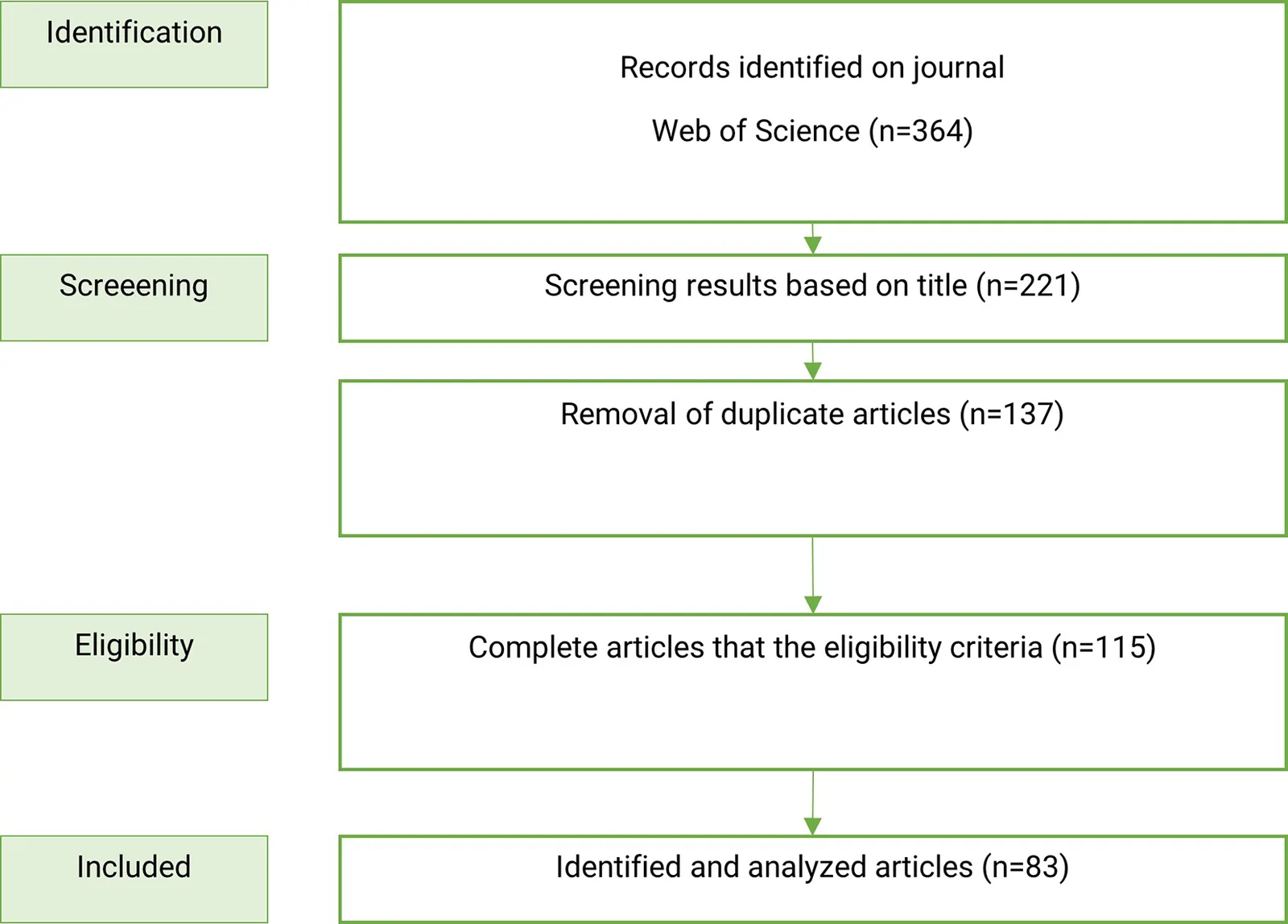
Prisma reporting.

Theoretical sampling continued until saturation and completeness were achieved, which was defined as reaching a sufficient number of research articles that identified conceptualizations pertinent to entrepreneurial leadership research. In total, 83 relevant research articles contributed to identifying and explaining these conceptualizations. Analyzing articles to elucidate entrepreneurial leadership conceptualizations (see Figure 1) necessitates constant comparison ( Helden et al., 2023). This process involves comparing codes and concepts to uncover and document their relationships, thereby refining labeled conceptualizations. These emergent conceptualizations serve as frameworks for subsequent article selection, guiding systematic deductions to select further articles. Memos are utilized to record emerging ideas and guide the selection of subsequent article samples. Each sampled article is scrutinized to discern its core contributions regarding the essence of entrepreneurial leadership or pivotal components within the conceptual framework of variables ( Helden et al., 2023) pertinent to entrepreneurial leadership research. Only after thorough analysis does the transition occur to selecting another article for examination.

## Results

**Figure 2.**
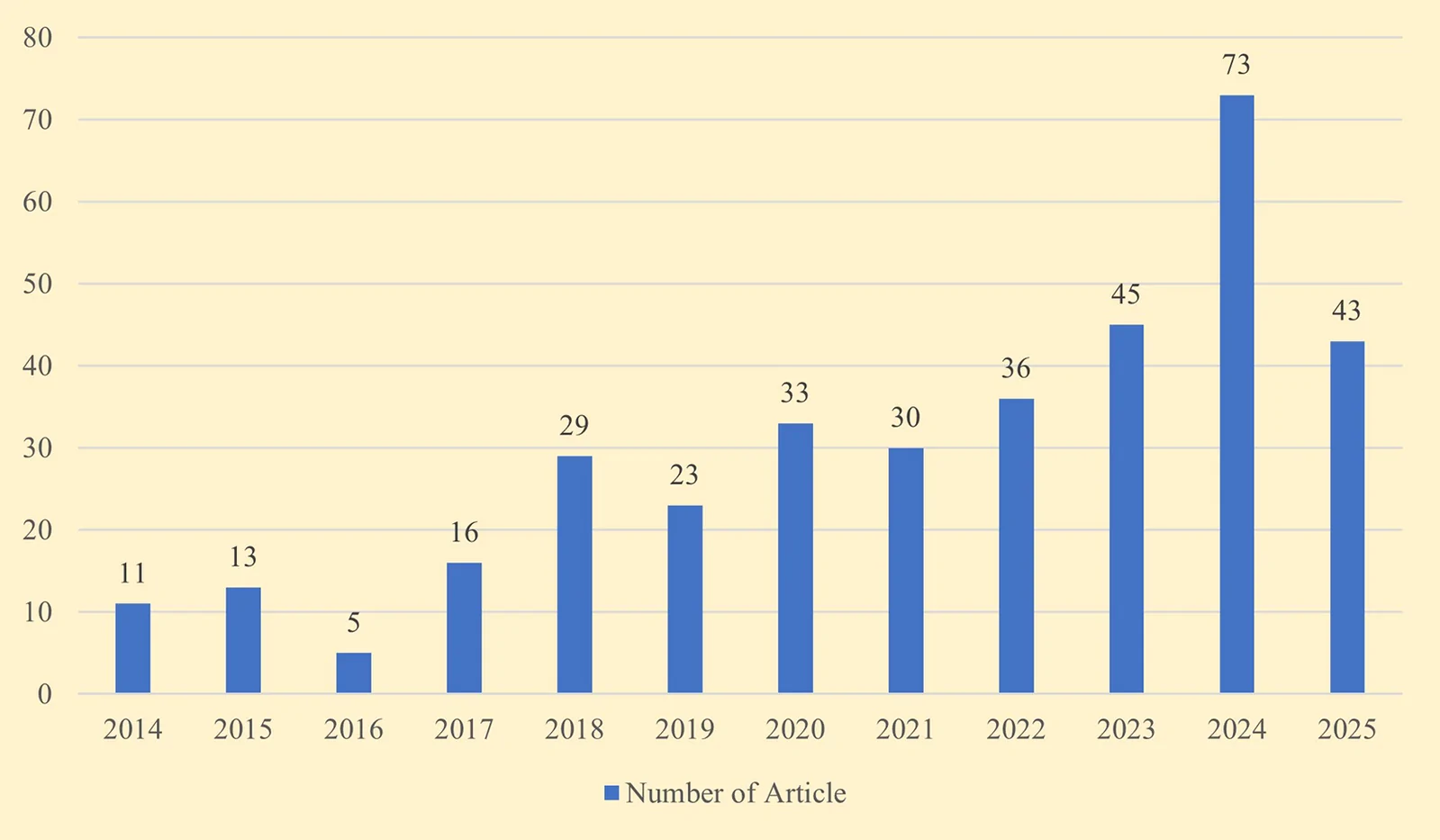
Evolution in the number of articles published.

The results bibliometric analysis starting from information in Figure 2 illustrates the trend in the number of articles published from 2014 to 2025. In 2014, 11 articles were published, and this number slightly increased to 13 in 2015. However, there was a significant drop in 2016, with only 5 articles published. Starting in 2017, the number of articles began to rise steadily, reaching 16 articles that year and then jumping to 29 in 2018. In 2019, there was a slight decrease to 23 articles, followed by an increase to 33 in 2020. In 2021, the number of publications declined slightly to 30 articles but increased again in 2022 to 36 articles, and further to 45 in 2023. The highest number of articles was recorded in 2024, with a total of 73. Although there was a slight decrease in 2025 to 43 articles, the publication volume remained significantly higher compared to the earlier years.

**Figure 3.**
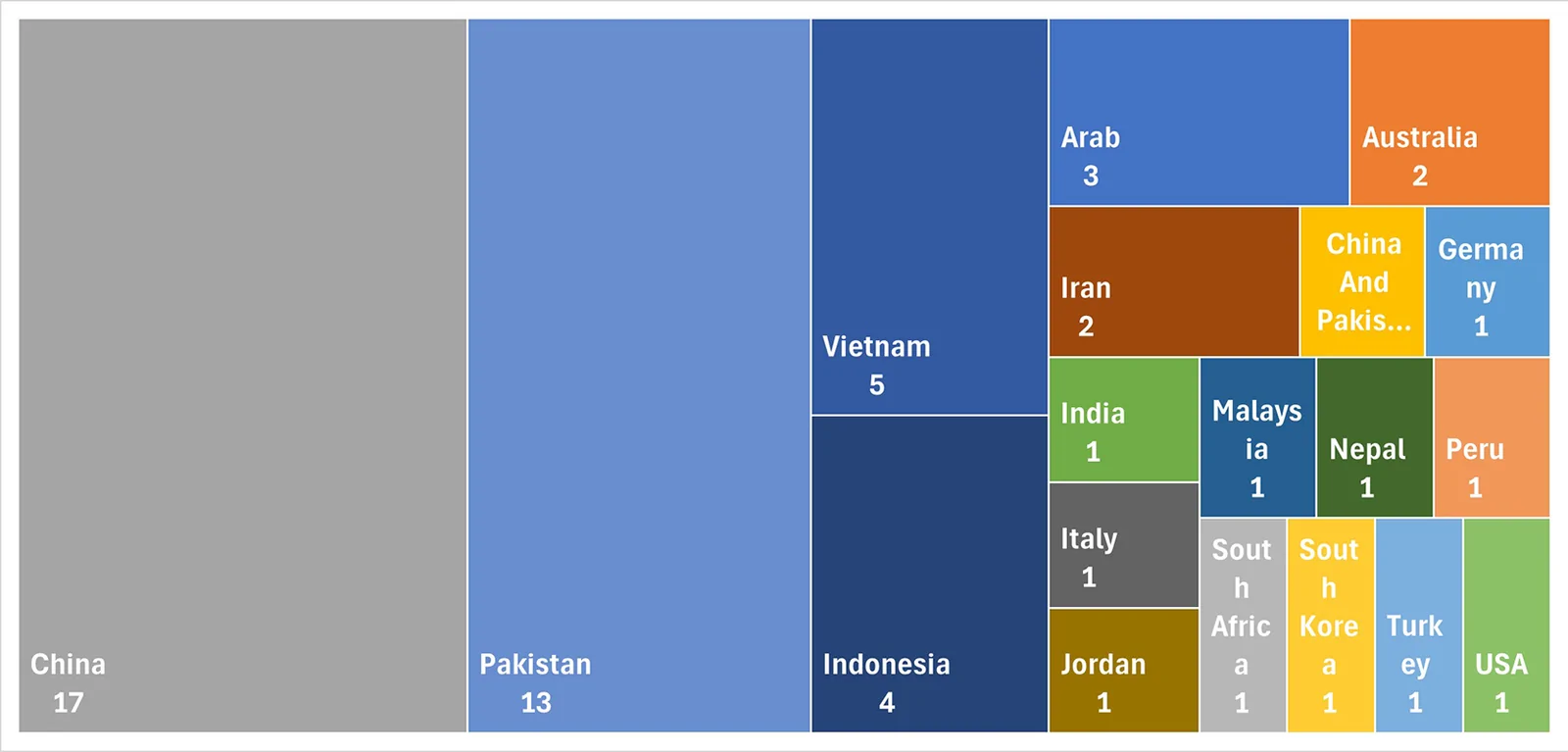
Tree map country classification.

Figure 3 is a tree map showing the classification of countries based on a specific size. In this treemap, each rectangle represents a single country, with the size of the rectangle indicating the magnitude of the value or amount associated with that country. The country of China has the largest rectangle, followed by Pakistan and Vietnam, each of which has a significant size. Indonesia also occupies a sizable area on this map. Other countries shown, albeit smaller in size, include Iran, Australia, India, South Africa, Saudi Arabia, Turkey, Italy, Nepal, USA, Jordan, Peru, Arabia, Germany, and South Korea. This treemap provides an effective visualization of the comparison between different countries in the context of their size or contribution to a given metric. This allows the reader to quickly understand the distribution and proportions between the countries involved.

Table 1 shows the ten institutions with the most relevant affiliations based on the number of publications related to entrepreneurial leadership research. The listed institutions are spread across different regions of the globe, demonstrating an international contribution to this research topic. The University of Tehran in Iran, Swinburne University of Technology in Australia, and the University of the West of Scotland in Southwest Scotland each lead with a total of 7 publications. The geographical distribution of these affiliations shows that entrepreneurial leadership research has received widespread attention from different countries and continents, with significant contributions from institutions in Iran, Australias, Scotland, China, the United Kingdom, Indonesia, and Vietnam.

**Table 1.** Top 10 most relevant affiliations.

No	Affiliation	Region	Number of publication
1	University of Tehran	Iran	7
2	Swinburne University of Technology	Australia	7
3	University of the West of Scotland	South-Western Scotland	7
4	Deakin University	Australia	6
5	University of Science and Technology Beijing	China	5
6	Nottingham Trent University	English	4
7	Universitas Padjadjaran	Indonesia	4
8	RMIT University Vietnam	Vietnam	4
9	Foreign Trade University	Vietnam	4
10	Xi'an Jiaotong University	China	4

Table 2 provides insight into the journals that cite articles related to entrepreneurial leadership research, particularly those published in journals indexed by Web of Science. Sustainability is 184 citations from 9 articles, showing significant influence in this area. The European Journal of Innovation Management, recorded 146 citations from 5 articles, while Cogent Business & Management had 37 citations from 5 articles. The Journal of Small Business Management stands out with the highest number of citations, reaching 548 citations from 4 articles, signifying its great relevance in entrepreneurial leadership research. Frontiers in Psychology and Leadership & Organization Development Journal, have 11 and 72 citations from 3 articles, respectively.

**Table 2.** Journal citation.

No	Journal	Tier	Citation	Total artikel
1	Sustainability	1	184	9
2	European Journal of Innovation Management	1	146	5
3	Cogent Business & Management	2	37	5
4	Journal of Small Business Management	1	548	4
5	Frontiers in Psychology	1	11	3
6	Leadership & Organization Development Journal	1	72	3
7	European Business Review	1	4	2
8	World Journal of Entrepreneurship, Management and Sustainable Development	1	51	2
9	Creativity And Innovation Management	1	109	2
10	Kybernetes	2	9	2
11	International Journal of Innovation Management	2	36	2
12	Journal of Business Research	1	253	2
13	Psychology Research and Behavior Management	2	55	2
14	Journal of Small Business and Enterprise Development	1	37	2
15	Journal of Service Theory and Practice	1	2	1
16	Journal of Services Marketing	1	15	1
17	Benchmarking-An International Journal	1	8	1
18	Journal of Manufacturing Technology Management	1	2	1
19	Management Decision	1	0	1

Table 3 shows the research variables that most frequently appear in studies related to entrepreneurial leadership, along with the number of studies that mention those variables and their researchers. The most dominant variable is Entrepreneurial Leadership, with 56 studies contributing, among them Awad et al., 2024a; Ercantan et al., 2024; Norena-Chavez & Romani Torres, 2024. This variable was followed by Employee Creativity and Entrepreneurial Leadership, which were mentioned in 5 studies each. Researchers such as Hou et al., 2024c Yang & Bentein, 2023 are some of the researchers who are researching Employee Creativity, while Hoang, Nguyen, et al. 2023b; Sultan et al., 2023 are researching Entrepreneurial Leadership.

**Table 3.** Research variables.

No	Variable	Total	Author
1	Entrepreneurial Leadership	61	Ahmed & Lucianetti, 2024; Ataei et al., 2024a; Awad et al., 2024; Ercantan et al., 2024; Getaneh Kebede et al., 2024; Hensellek et al., 2023; Hou et al., 2024a; G. Hussain et al., 2023; Khan et al., 2024; Norena-Chavez & Romani Torres, 2024; Taleb et al., 2023; Tariq et al., 2024; Zahoor et al., 2023; Abualoush et al., 2022; Chingwena & Scheepers, 2022; Djalil et al., 2023; Hai et al., 2021; Haq & Aslam, 2023; Hoang et al., 2022a, 2024; N. Hussain & Li, 2022; Pu, Ji, et al., 2022a; Yang & Bentein, 2023; Zhang et al., 2023; Alsharif et al., 2021; Bilal et al., 2021; Islam & Asad, 2024; Li et al., 2020; Mehmood, Jian, & Gilal, 2020b, Mehmood et al., 2022; Rehman et al., 2021; Wu et al., 2021; Bagheri & Harrison, 2020; Imran & Aldaas, 2020; Iqbal et al., 2022; Latif et al., 2020; Musara & Nieuwenhuizen, 2020; Wahab & Tyasari, 2020; Bagheri & Akbari, 2018; Li et al., 2020; Newman et al., 2018a; Paudel, 2019; Soomro et al., 2019; Utoyo et al., 2020; Yang et al., 2019a; Akbari et al., 2021; Fontana & Musa, 2017; Hoang et al., 2023b; Huang et al., 2014; Lin & Yi, 2023; Mehmood, Jian, & Gilal, 2020b; Sultan et al., 2023.
2	Employee Creativity	5	Cai et al., 2019; Hou et al., 2024b; Islam & Asad, 2024; Ximenes et al., 2019; Yang & Bentein, 2023
3	Project Success	4	Ahmed & Lucianetti, 2024; Latif et al., 2020; Norena-Chavez & Romani Torres, 2024; Tariq et al., 2024
4	Creative Self-efficacy	3	Akbari et al., 2021; Mehmood, Jian, & Akram, 2020a; Sultan et al., 2023
5	Entrepreneurial Intentions	3	G. Hussain et al., 2023; Khan, 2022; Shabeer, 2023
6	Innovative Behavior	6	Bagheri & Harrison, 2020; Ercantan et al., 2024; Hoang et al., 2022b; Iqbal et al., 2022; Mehmood, Jian, & Akram, 2020a; Newman et al., 2018a
7	Intrinsic Motivation	3	Hoang et al., 2022c; Sultan et al., 2023
8	Knowledge Sharing	3	Abualoush et al., 2022; Islam et al., 2024; Khawaldeh & Alzghoul, 2024
9	Team Creativity	3	Cai et al., 2019; Hoang et al., 2022b; Mehmood et al., 2022
10	Affective Commitment	2	Pu, Sang, et al., 2022b; Yang et al., 2019b
11	Competitive Advantage	2	Ercantan et al., 2024; Hoang et al., 2022b
12	Creative Self Efficacy	2	Cai et al., 2019; Newman et al., 2018b
13	Entrepreneurial Orientation	2	Ataei et al., 2024b; Nguyen et al., 2021
14	Entrepreneurial Self-Efficacy	2	G. Hussain et al., 2023; Yang & Bentein, 2023
15	Entrepreneurial Success	2	N. Hussain & Li, 2022; Taleb et al., 2023
16	Idea Generation	2	Bagheri & Akbari, 2018; Fontana & Musa, 2017
17	Innovation Performance	2	Fontana & Musa, 2017; Utoyo et al., 2020
18	Innovative Work Behavior	2	Abualoush et al., 2022; Li et al., 2020
19	Big Data Capability	1	Tariq et al., 2024
20	Entrepreneurial Bricolage	1	Ahmed & Lucianetti, 2024
21	Knowledge Infrastructure Capability	1	Tariq et al., 2024
22	Knowledge Management Processes	1	Latif et al., 2020
23	Knowledge Based Dynamic Capability	1	Tariq et al., 2024
24	Team Entrepreneurial Passion	1	Norena-Chavez & Romani Torres, 2024
25	Team Innovation Culture	1	Norena-Chavez & Romani Torres, 2024
26	Team Reflexivity	1	Norena-Chavez & Romani Torres, 2024

Table 4 shows the various theories that are often used in research related to entrepreneurial leadership, complete with the number of citations and the number of articles supporting the theory, as well as the names of relevant researchers. Social Cognitive Theory digunakan oleh 19 peneliti terdahulu dengan sitasi terbanyak yaitu 714. Teori yang digunakan terbanyak kedua adalah Resource Based View Theory. Resource Based View Theory digunakan sebanyak 14 peneliti terdahulu dengan jumlah sitasi 163. Social Learning Theory memiliki sitasi sebanyak 298 dengan 10 peneliti. Dynamic Capability Theory disitasi sebanyak 35 kali, Entrepreneurial Leadership Theory disitasi sebanyak 117 kali dan Self-Determination Theory SDT disitasi sebanyak 28 kali. Empat peneliti berbeda melakukan penelitian dengan theory Social Exchange Theory, Theory Of Planned Behavior, dan Upper Echelons Theory. Contingency Theory, Effectuation Theory, Knowledge-Based View KBV Theory, Resource Dependence Theory, Social Identity Theory, Social Information Processing Theory masing masing digunakan oleh 2 artikel yang berbeda.

**Table 4.** Report theory citation.

No	Theory	Citation	Total article	Authors
1	Social Cognitive Theory	714	19	Getaneh Kebede et al., 2024; Hoang et al., 2023b; Li et al., 2020; Rehman et al., 2021; Wahab & Tyasari, 2020; Yang & Bentein, 2023; Zahoor et al., 2023; Akbari et al., 2021; Awad et al., 2024; Bagheri & Harrison, 2020; Cai et al., 2019; Hoang et al., 2022b; Malibari & Bajaba, 2022; Newman et al., 2018b; G. Hussain et al., 2023; Iqbal et al., 2022; Megheirkouni et al., 2020; Sultan et al., 2023
2	Resource Based View Theory	163	14	Abdullah Alshammari et al., 2023; Al Mamun et al., 2018; Ercantan et al., 2024; Getaneh Kebede et al., 2024; Hai et al., 2021; Khan et al., 2024b; Norena-Chavez & Romani Torres, 2024; Razzaque et al., 2024; Taleb et al., 2023; Tariq et al., 2024; Wahab & Tyasari, 2020; Wu et al., 2021
3	Social Learning Theory	298	10	Dixit et al., 2023; Iqbal et al., 2022; Islam et al., 2024; Lee et al., 2022; Mehmood, Jian, & Gilal, 2020b; Miao et al., 2019; Newman et al., 2018b; Norena-Chavez & Romani Torres, 2024; Yang & Bentein, 2023
4	Dynamic Capability Theory	35	5	Haq & Aslam, (2023). Hoang, Luu, et al., 2023a; Hoang, Nguyen, et al., 2023b; Tariq et al., 2024; Utoyo et al., 2020
5	Entrepreneurial Leadership Theory	117	5	Bagheri & Harrison, 2020; Iqbal et al., 2022; Rehman et al., 2021; Sultan et al., 2023; Yang et al., 2019b
6	Self-Determination Theory	28	5	Bilal et al., 2021, 2022; Li et al., 2020; Pu, Sang, et al., 2022b; Sipahi Dongul & Artantaş, 2023; Zhang et al., 2023
7	Social Exchange Theory	158	4	Iqbal et al., 2022; Islam & Asad, 2024; Newman et al., 2018b; Pu, Sang, et al., 2022b
8	Theory Of Planned Behavior	3	4	Dhakal et al., 2022; G. Hussain et al., 2023; Khan et al., 2024; Zhang et al., 2023
9	Upper Echelons Theory	67	4	Ali & Ghildiyal, 2023; Hensellek et al., 2023; Nor-Aishah et al., 2020; Zahoor et al., 2023
10	Contingency Theory	21	2	Shabeer, 2023; Wahab & Tyasari, 2020
11	Effectuation Theory	59	2	Ahmed & Lucianetti, 2024; Nor-Aishah et al., 2020
12	Knowledge-Based View KBV Theory	9	2	N. Hussain & Li, 2022; Tariq et al., 2024
13	Resource Dependence Theory	43	2	Hai et al., 2021; Hoang, Luu, et al., 2023a
14	Social Identity Theory	33	2	Lee et al., 2022; Musara & Nieuwenhuizen, 2020
15	Social Information Processing Theory	108	2	Iqbal et al., 2022; Miao et al., 2019
16	Absorptive Capacity Theories	30	1	Wu et al., 2021
17	Cognitive-affective Personality System Theory	8	1	Pu, Sang, et al., 2022b
18	Collective Identity Theory	32	1	Musara & Nieuwenhuizen, 2020
19	Complex Adaptive System Theory	3	1	Lin & Yi, 2023
20	Conservation Of Resources Theory	0	1	Hou et al., 2024b
21	Creativity Theory	8	1	Dixit et al., 2023
22	Culturally Endorsed Leadership Theories	65	1	Miao et al., 2019
23	Empowerment Theory	8	1	Dixit et al., 2023
24	Entrepreneurial Event Theory	0	1	Shabeer, 2023
25	Entrepreneurial Vision	65	1	Miao et al., 2019
26	Entrepreneurship and Innovation Theory	29	1	Utoyo et al., 2020
27	Explorer Behavior EXPB	5	1	Rehman et al., 2021
28	Gender Theory	4	1	Megheirkouni et al., 2020
29	Institutional Theory	110	1	Yousafzai et al., 2015
30	Instrumental Stakeholder Theory	5	1	Sipahi Dongul & Artantaş, 2023
31	Intrinsic Motivation Theory	8	1	Dixit et al., 2023
32	Job Embeddedness Theory	1	1	Pu, Sang, et al., 2022b
33	Organization Learning Theory	31	1	Islam et al., 2024
34	Person-Environment Fit Theory	0	1	Shabeer, 2023
35	Self-Efficacy Theory	47	1	Li et al., 2020
36	Social Capital Theory	5	1	Sipahi Dongul & Artantaş, 2023
37	Social Construction Theory	4	1	Megheirkouni et al., 2020
38	Social Role Theory	30	1	Yang et al., 2019a
39	Strategic Entrepreneurship Theory	1	1	Zahoor et al., 2023
40	Technology Affordances and Constraints Theory	30	1	Wu et al., 2021
41	The Affective Events Theory	0	1	Hou et al., 2024b
42	Theory Of Creativity	2	1	Sultan et al., 2023

In addition, the theories used in conducting EL research are Absorptive Capacity Theories, Cognitive-affective Personality System Theory, Collective Identity Theory, Complex Adaptive System Theory, Conservation Of Resources Theory, Creativity Theory, Culturally Endorsed Leadership Theories, Empowerment Theory, Entrepreneurial Event Theory, Entrepreneurial Vision, Entrepreneurship and Innovation Theory, Explorer Behavior EXPB, Gender Theory, Institutional Theory, Instrumental Stakeholder Theory, Intrinsic Motivation Theory, Job Embeddedness Theory, Organization Learning Theory, Person-Environment Fit Theory, Psychological Theories, Self-Efficacy Theory, Social Capital Theory, Social Construction Theory, Social Role Theory, Strategic Entrepreneurship Theory, Technology Affordances and Constraints Theory, The Affective Events Theory, Theory Of Creativity. Although only 1 article wrote each of these theories, there are theories that are cited many times, namely Social Information Processing Theory 108 times and Institutional Theory 110 times.

In Table 5, Resource-Based View Theory is used to base the research variables Accountability, Analytical Thinking, Competitive Advantage, Corporate Sustainable Development, Emotional Intelligence, Entrepreneurial Leadership, Entrepreneurial Leadership Skills, Entrepreneurial Opportunity Recognition, Entrepreneurial Success, Firm Performance, Innovation Capability, Innovative Behaviour, Micro-Entreprise Performance, Micro-Enterprise Sustainability, People-Oriented Leadership Style, Responsibility, Saudi Female Entrepreneurs, Task-Oriented Leadership Style ( Abdullah Alshammari et al., 2023; Al Mamun et al., 2018; Ercantan et al., 2024; Razzaque et al., 2024; Taleb et al., 2023). These resources allow companies to create strategies that are difficult for competitors to replicate ( Barney, 1991). RBV views the company as a collection of historical and "semi-permanent" resources, including physical capital, brand names, and organizational routines and capabilities. These resources are the basis for competitive advantage ( Lockett *et al*., 2009).

**Table 5.** Top 10 most research variables from theory.

No	Theory	Variable	Author
1	Resource-Based View Theory	a. Accountability b. Analytical Thinking c. Competitive Advantage d. Corporate Sustainable Development e. Emotional Intelligence f. Entrepreneurial Leadership g. Entrepreneurial Leadership Skills h. Entrepreneurial Opportunity Recognition i. Entrepreneurial Success j. Firm Performance k. Innovation Capability l. Innovative Behaviour m. Micro-Entreprise Performance n. Micro-Enterprise Sustainability o. People-Oriented Leadership Style p. Responsibility q. Saudi Female Entrepreneurs r. Task-Oriented Leadership Style	Abdullah Alshammari et al., 2023; Al Mamun et al., 2018; Ercantan et al., 2024; Razzaque et al., 2024; Taleb et al., 2023
2	Social Cognitive Theory SCT	a. Creative Self Efficacy b. Creative Self-efficacy c. Employee Creativity d. Employees` Innovative Behaviour e. Employees` Intellectual Agility f. Entrepreneurial Leadership g. Entrepreneurial Leadership h. Ethical Leadership i. Individual Creativity Self Efficacy j. Innovation Climate k. Innovation Work Behavior l. Innovation Work Behaviour m. Innovative Behavior n. Innovative Behaviour o. Entrepreneurial Leadership p. Intrinsic Motivation q. Proactive Work Behavior r. Service Innovative Behavior s. Support For Innovation t. Team Creative Efficacy u. Team Creativity v. Team Creativity Self-efficacy w. Trush In Leader	Awad et al., 2024; Bagheri & Harrison, 2020; Cai et al., 2019; Hoang et al., 2022a; Malibari & Bajaba, 2022; Newman et al., 2018b
3	Complex Adaptive System Theory	a. Adaptive Innovation b. Boundary-Spanning Integration c. Entrepreneurial Leadership d. Exploitative Learning e. Exploratory Learning f. Resource Bricolage	Lin & Yi, 2023
4	Dynamic Capability Theory	a. Entrepreneurial Leadership b. Supply Chain Performance c. Supply Chain Resilience	Haq & Aslam, 2023
5	Knowledge-Based View KBV Theory	a. Entrepreneurial Leadership b. Entrepreneurial Success c. Knowledge Management Processes	N. Hussain & Li, 2022
6	Self-Determination Theory SDT	a. Entrepreneurial Leadership b. Entreprenurial Leadership c. Proactive Work Behavior d. Proactive Work Behavior e. Psychological Well-Being f. Sustainable Employability g. Work Uncertainty	Bilal et al., 2021, 2022
7	Social Exchange Theory SET	a. Employee Creativity b. Entrepreneurial Leadership c. Knowledge Sharing	Islam & Asad, 2024
8	Social Learning Theory	a. Employee Creative b. Entrepreneurial Leadership c. Entrepreneurial Leadership d. Team Creativity	Mehmood, Jian, & Akram, 2020a
9	Theory Of Planned Behavior	a. Entrepreneurial Leadership b. Entrepreneurial Passion c. Venture Growth Intentions	Dhakal et al., 2022
10	Upper Echelons Theory	a. Entrepreneurial Leadership b. Green Innovation c. Organizational Learning Culture d. Strategic Flexibility e. Venture Performance	Ali & Ghildiyal, 2023; Hensellek et al., 2023

**Figure 4.**
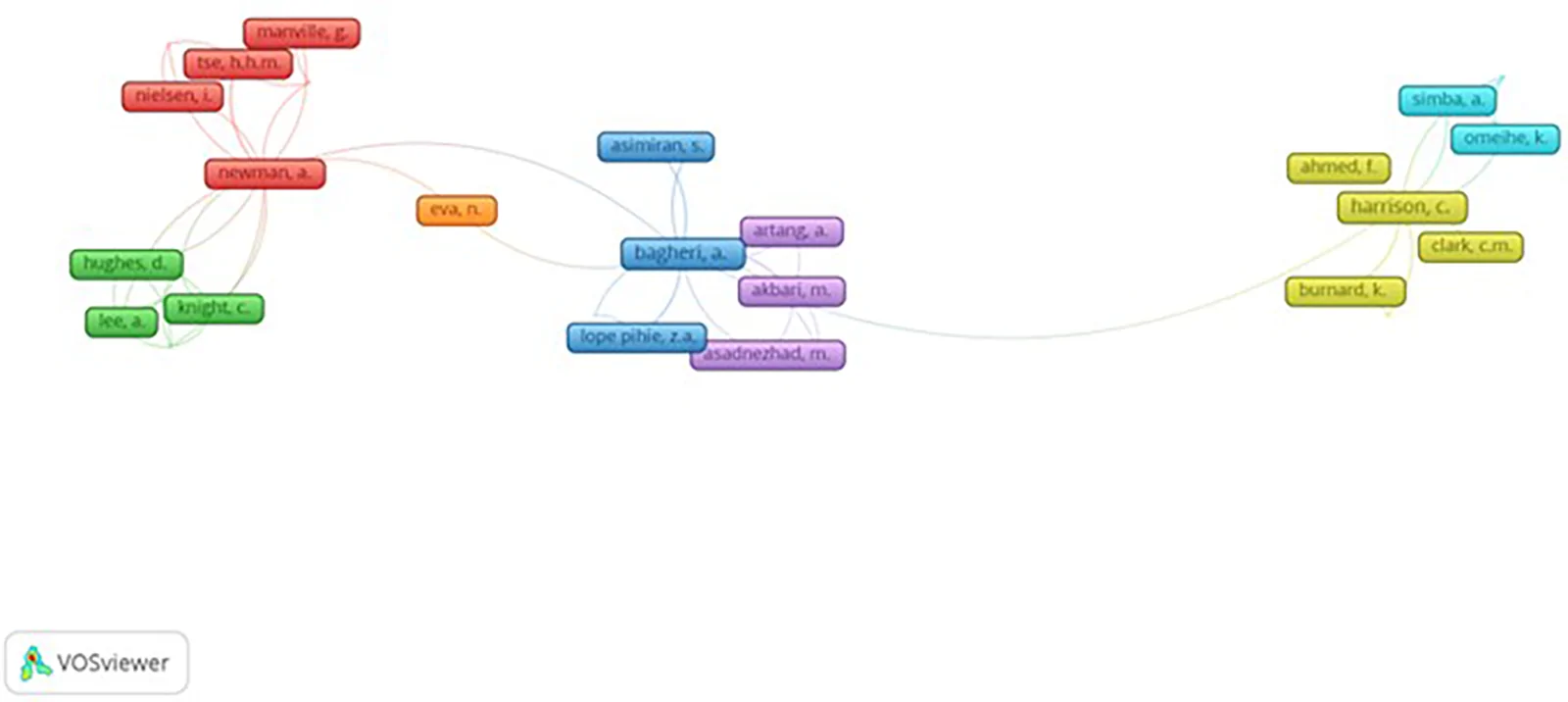
Graphical representation of authors’ network.

Graphical information from the authors' network with the theme of Entrepreneurial Leadership was created using VOSviewer in figure 4. Each color group in this diagram shows a cluster of authors who often work together, and some authors act as liaisons between those groups. The group of authors in red has Newman, A. as its center, suggesting that this author has many collaborations with other authors in the group, such as Manville, G., Tse, H.H.M., and Nielsen, I. These writers form a close network with strong connections between them. On the other hand, the group of authors in green is made up of Hughes, D., Lee, A., and Knight, C., which also shows significant collaboration among them, albeit on a smaller scale than the red group.

The orange group of authors consists only of Eva, N., who was connected to the red group through a collaboration with Newman, A. This suggests that although Eva N. does not collaborate well within her own group, she still plays an important role in the overall network through her connection with Newman, A. Meanwhile, the group of writers in blue is led by Bagheri, A., who has many collaborations with writers such as Asimiran, S., Lope Pihie, Z.A., Artang, A., Asadnezhad, M., and Akbari, M. This group shows a strong and interconnected collaboration dynamic. On the other hand, the group of authors in yellow consists of Burnard, K., Clark, C.M., Harrison, C., and Ahmed, F., which also shows close collaboration between them. The group was connected to the cyan-colored writers' group, consisting of Simba, A. and Omeihe, K., through a collaboration involving several writers from both groups. This diagram provides a clear visual picture of how these networks of authors are interconnected and demonstrates the importance of collaboration in academic research.

Table 6 provides an overview of Entrepreneurial Leadership Characteristics influence on Business Organization Outcomes. This table shows that the variables Individual Capability, Team Behaviour, Entrepreneurial Bricolage will affect Project Success. Career Growth Opportunities have an effect on Entrepreneurial performance. Entrepreneurial Opportunity Recognition, Knowledge Management Processes, Innovation Capability have an impact on Entrepreneurial success. Employee Creativity, Intrinsic Motivation will create New product development performance. Ambidextrous Learning, Ethical Behaviour, Perceived Organizational Support, Proactiveness, Risk Taking, Social Entrepreneurial encourage the improvement of Organizational performance. Subsidiary Organizational Inertia, Subsidiary Decision Autonomy, Subsidiary Task Complexity determine Subsidiary performance. Risk taking, Entrepreneurial Competencies, Entrepreneurial intentions can improve Enterprise performance.

**Table 6.** Entrepreneurial leadership influence on employee characteristic.

Categories (Themes)	Outcomes	Exemplary papers
Individual Capability, Team Behaviour, Entrepreneurial Bricolage	Project Success	Ahmed & Lucianetti, 2024; Latif et al., 2020; Norena-Chavez & Romani Torres, 2024; Tariq et al., 2024
Career Growth Opportunities	Entrepreneurial performance	Pu, Ji, et al., 2022a
Entrepreneurial Opportunity Recognition, Knowledge Management Processes, Innovation Capability	Entrepreneurial success	Hussain & Li, 2022; Taleb et al., 2023
Employee Creativity, Intrinsic Motivation	New product development performance	Sultan et al., 2023
Ambidextrous Learning, Ethical Behaviour, Perceived Organizational Support, Proactiveness, Risk Taking, Social Entrepreneurial	Organizational performance	Imran & Aldaas, 2020; Sarmawa et al., 2020; Sipahi Dongul & Artantaş, 2023; Wu et al., 2021
Subsidiary Organizational Inertia, Subsidiary Decision Autonomy, Subsidiary Task Complexity	Subsidiary performance	Sarabi et al., 2020
Risk Taking, Entrepreneurial Competencies, Entrepreneurial intentions	Enterprise performance	Khan et al., 2024

**Figure 5.**
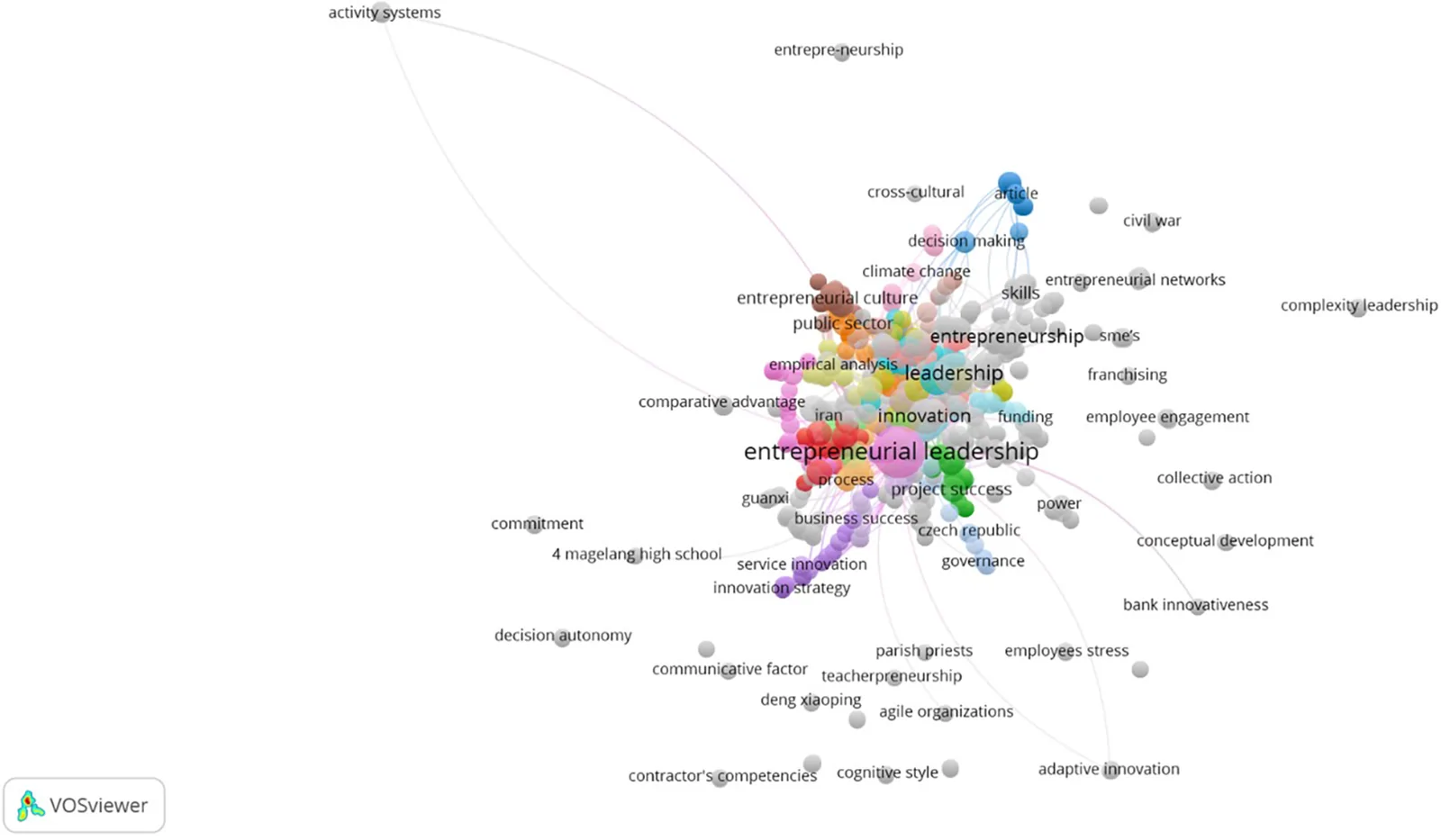
Graphical representation of co-occurrence network (frames network visualization).

Network visualization is seen in figure 5. The most widely used keyword is entrepreneurial leadership according to the topic carried out by the author. Keywords related to individual analysis units consist of innovatice work behavior, creative self efficacy, ethical entrepreneurial leader, absorptive capability, entrepreneurial bricolage, and adaptive innovation. Meanwhile, keywords related to organizational analysis units consist of innovation performance. In this network visualization, it also displays information on keyword theory that are often used in articles is upper echelons theory. The leadership styles that are often related to the topic of EL. Agriculture students are also quite interesting topics in EL's research. It is possible for Agriculture students to have a unique character in providing leadership in an organization. The countries that are often used to conduct EL research is African.

Table 7 shows the difference between Employee Skills and Entrepreneurial Leadership Factors. To achieve sustainable competitive advantage, technical/business skills are required to achieve Big Data Analytic Capilibity, Knowledge-based Dynamic Capability, Infrastructure Capability, Dynamic Capabilities. Conceptual Skills are needed to support Product Innovation, Know-Source Management Processes, Green Innovation Behavior, Idea Generation, Strategic Flexibility, Design Thinking, Employees Intellectual Agility, and Supply Chain Resilience. Interpersonal skills focus on Innovation Work Behaviour, Employee Creativity, Proactive Work Behavior, Tacit Knowledge Sharing, Entrepreneurial Intentions, Intrinsic Motivation, and Affective Commitment. The most important skills to have are Entrepreneurial skills to create Entrepreneurial Passion, Entrepreneurial Opportunity Recognition, Individual Creativity, Self Efficacy, and Entrepreneurial Resilience.

**Table 7.** Entrepreneurial leadership factors and their exemplary papers.

Categories (Themes)	Entrepreneurial leadership factors	Exemplary papers
Technical/business skills	1. Big Data Analytic Capilibity 2. Knowledge-based Dynamic Capability 3. Knowledge 4. Infrastructure Capability 5. Dynamic Capabilities	Nguyen et al., 2021; Tariq et al., 2024
Conceptual skills	1. Product Innovation 2. Knowlegde Management Processes 3. Green Innovation Behavior 4. Idea Generation 5. Strategic Flexibility 6. Design Thinking 7. Employees` Intellectual Agility 8. Supply Chain Resilience	Ali & Ghildiyal, 2023; Bagheri & Akbari, 2018; Fontana & Musa, 2017; Haq & Aslam, 2023; Hensellek et al., 2023; Hoang, Nguyen, et al., 2023b; N. Hussain & Li, 2022; Malibari & Bajaba, 2022; Rehman et al., 2021
Interpersonal skills	1. Innovation Work Behaviour 2. Employee Creativity 3. Proactive Work Behavior 4. Tacit Knowledge Sharing 5. Entrepreneurial Intentions 6. Intrinsic Motivation 7. Affective Commitment	Abualoush et al., 2022; Awad et al., 2024; Bagheri, Akbari, et al., 2022a; Bagheri, Newman, et al., 2022b; Bilal et al., 2022; O. Ercantan & Eyupoglu, 2022; Hoang, Nguyen, et al., 2023b; G. Hussain et al., 2023; Iqbal et al., 2022; Islam et al., 2024; Khan et al., 2024; Li et al., 2020; Mehmood, Jian, & Akram, 2020a; Newman, Tse, et al., 2018a; Pu, Ji, et al., 2022a; Shabeer, 2023; Yang et al., 2019a; Zhang et al., 2023
Entrepreneurial skills	1. Entrepreneurial Passion 2. Entrepreneurial Opportunity Recognition 3. Individual Creativity Self Efficacy 4. Entrepreneurial Resilience	Dhakal et al., 2022; Khan et al., 2024; Taleb et al., 2023

Table 8 outlines the Outcomes of the Entrepreneurial Leadership Model. Individual outcomes in Entrepreneurial Leadership research consist of Big Data Analytic Capilibity, Knowledge-based Dynamic Capability, Knowledge Infrastructure Capability, Dynamic Capabilities, Product Innovation, Knowlede Management Processes, Green Innovation Behavior, Idea Generation, and Employees' Intellectual Agility. In addition, Entrepreneurial Leadership research also has an impact on team outcomes in the form of Team Creativity, Team Reflexivity, Team Innovation Culture, and Team Entrepreneurial Passion. Organizational Outcomes from Entrepreneurial Leadership research are in the form of Project Success, Entrepreneurial Performance, Entrepreneurial Success, Supply Chain Performance, Business Performance, Bank Performance, and Job Performance.

**Table 8.** Features of the entrepreneurial leadership model.

Individual outcomes	Team outcome	Organizational outcomes
1. Big Data Analytic Capilibity 2. Knowledge-based Dynamic Capability 3. Knowledge Infrastructure Capability 4. Dynamic Capabilities 5. Product Innovation 6. Knowlegde Management Processes 7. Green Innovation Behavior 8. Idea Generation 9. Employees` Intellectual Agility	1. Team Creativity 2. Team Reflexivity 3. Team Innovation Culture 4. Team Entrepreneurial Passion	1. Project Success 2. Entrepreneurial Performance 3. Entrepreneurial Success 4. Supply Chain Perfomance 5. Business Performance 6. Bank Performance 7. Job Performance

## Discussion

Social Cognitive Theory (SCT) describes how individuals shape behavior through interactions between environment, experience, and cognition. In this context, this theory is widely used to explain innovative and proactive behavior in organizations. Bilal et al., (2021) states that proactive work behavior is influenced by self-perception, past experiences, and future expectations that are in accordance with the observation and reinforcement mechanisms in the SCT. Employees with high self-confidence and social support tend to exhibit initiative and anticipatory behaviors. Bagheri et al., (2022b); Cai et al., (2019); Newman, Neesham, et al., (2018); Sultan et al., (2023) emphasizing that an individual's belief in his or her creative self-efficacy is a central aspect of SCT. This belief encourages individuals to try new approaches, solve problems innovatively, and not be afraid to fail. Sultan et al., (2023) Show that intrinsic motivation (the internal drive to do something because it is fun or meaningful) is aligned with self-efficacy beliefs and personal values in SCT. Ercantan et al., (2024); Hoang et al., (2022); Iqbal et al., (2022) concludes that innovative behaviors are the result of a combination of self-efficacy, observational learning, and a work environment that supports experimentation. SCT describes how individuals model the behavior of innovative colleagues.

Latif et al., (2020) argues that knowledge management processes (such as acquisition, sharing, and deployment) are core components in improving organizational innovation capabilities. KBV emphasizes that organizations must create a system that allows the effective use of knowledge. N. Hussain & Li, (2022); Taleb et al., (2023) highlighting that entrepreneurial success is heavily influenced by the way knowledge is developed and managed. KBV explained that the combination of explicit and tacit knowledge is the foundation in creating entrepreneurial value.

RBV explains that unique, unreplicable, and valuable internal resources are key to long-term competitive advantage. In this context, leadership and opportunity recognition are two forms of strategic human resources. Study by Abdullah Alshammari et al., (2023); Al Mamun et al., (2018); Ercantan et al., (2024); Getaneh Kebede et al., (2024); Hai et al., (2021); Khan et al., (2024); Norena-Chavez & Romani Torres, (2024); Razzaque et al., (2024); Taleb et al., (2023); Wahab & Tyasari, (2020); Wu et al., (2021) shows that entrepreneurial leadership is a strategic resource that affects business performance. RBV views this capability as a unique resource that allows organizations or individuals to pursue new opportunities before competitors.

## Conclusion

Research on entrepreneurial Leadership has seen significant growth in the last decade, with major contributions from developed countries such as the United States and leading organizations such as Harvard University. The journal "Journal of Business Venturing" emerged as the primary source for this study. The research focus that is often encountered includes innovation, organizational performance, and intrinsic motivation, with transformational leadership theory and intellectual capital as the dominant theoretical basis. In the future, research is expected to continue to develop with a focus on digitalization and globalization in the context of entrepreneurial Leadership. The areas of research that are anticipated to be the focus in the future include the role of entrepreneurial Leadership in the context of digitalization and globalization. The research is also expected to explore more deeply the impact of new technologies and innovative business models on entrepreneurial Leadership.

Research related to EL can also be done by comparing the conditions of two or more countries. EL is widely carried out in business organizations, it needs to be expanded in non-business sectors such as education and health organizations. There are still many limitations of the research conducted in this study. This research is primarily based on articles available in Scopus, which may not include all publications related to Entrepreneurial leadership, so there is a possibility of bias in the publications accessed. The EL variable can also be used as a moderation and mediation variable. This is because currently many people have conducted research using EL variables to be Variable Independent and Variable Dependent.

## Data Availability

Zenodo: Dataset: Entrepreneurial Leadership in Business Organizations: a systematic review and meta-analysis. https://doi.org/10.5281/zenodo.18425242. ( Amborowati, A., et al., 2026). This project contains the following underlying data:
1. Dataset_Entrepreneurial Leadership.xlsx 2. PRISMA flow diagram for Entrepreneurial Leadership.pdf 3.
PRISMA_Checklist_Literature Data Integration of Entrepreneurial Leadership.docx Data is available under the terms of the e Creative Commons Attribution 4.0 International license (CC-BY 4.0).
