# Entrepreneurial Leadership in Business Organizations: a systematic review and meta-analysis

**DOI:** 10.12688/f1000research.175169.3

**Published:** 2026-07-29

**Authors:** Agustin Amborowati, Asri Laksmi Riani, Mugi Harsono, Joko Suyono

**Affiliations:** 1 Departement of Business Management, Vocational School, Universitas Sebelas Maret, Surakarta, Central Java, 57126, Indonesia; 2 Departement of Management, Faculty Economic and Business, Universitas Sebelas, Maret, Surakarta, Central Java, 57126, Indonesia

**Keywords:** Entrepreneurial Leadership, Leadership, Leadership Styles, Leadership in Entrepreneurship, Systematic Literature Review

## Abstract

This systematic literature review provides a comprehensive analysis of the current state of research on entrepreneurial leadership over the past decade. The study addresses key research questions, including publication frequency, productive countries, organizations, and journals, most researched variables, and most cited theories and influential authors. PRISMA flowchart was used, comprising four main stages: identification, screening, eligibility, and inclusion. Initially, 364 articles were identified through database searches; at the eligibility stage, 83 articles underwent full-text review. Findings reveal a significant increase in publications on entrepreneurial leadership, with Indonesia emerging as the most productive country and the Journal of Small Business Management as the most cited journal. Key variables such as employee creativity, entrepreneurial leadership, project success, and creative self-efficacy are frequently studied, with Social Cognitive Theory and Social Learning Theory being prominent theoretical foundations. While the study offers valuable insights, it acknowledges certain limitations, including potential publication bias due to the reliance on specific databases and the underrepresentation of contributions from developing countries. This review not only identifies key themes and trends but also proposes a conceptual framework for further research and practical insights for business leaders and entrepreneurs.

## Introduction

Awad et al. (2024) conducted a cross-sectional study among undergraduate nursing students in Thailand, revealing that a significant number of students exhibit high levels of entrepreneurial leadership. The entrepreneurial characteristics of nursing education can improve students' skills and readiness for innovative healthcare practices. Clark et al., (2023) highlighting the implementation of entrepreneurial leadership (EL) in the public sector, EL plays a role in promoting the principles of democratic principles and can improve organizational effectiveness. Soomro et al., (2019) Observing entrepreneurial leadership behaviors among college students, they showed that activities that promote cognitive flexibility can significantly improve students' entrepreneurial leadership behaviors, thus preparing them for future leadership roles in diverse organizational settings.

An essential point in entrepreneurial leadership is to consider the concept of open innovation ( Ataei et al., 2024a). Research related to EL has been widely conducted, some focusing on individual units of analysis ( Abualoush et al., 2022; Cai et al., 2019; K. Ercantan et al., 2024; Hoang, Luu, et al., 2023a; Hou et al., 2024; Iqbal et al., 2022; Islam & Asad, 2024; Yang & Bentein, 2023) and organizations ( Khan et al., 2024; Paudel, 2019; Sarabi et al., 2020; Sarmawa et al., 2020; Sultan et al., 2023; Wu et al., 2021). This systematic literature review aims to explore research related to EL that has been carried out so far. This study synthesizes documents based on a systematic review in four stages, similar to the approach used by ( Hosany et al., 2022). However, it was further developed. The uncertain business environment makes organizations forced to continue to innovate and be able to adapt quickly to change. Entrepreneurial Leadership (EL) is able to combine opportunity recognition, innovation, and strategic decision-making in developing adaptive strategies, improving organizational performance, and being able to create a competitive advantage.

Previous studies of EL have been focused on various theoretical perspectives, contexts, and methodologies. Understanding of variables related to EL, EL in supporting innovation, and entrepreneurial leadership principles to achieve business success has not been explored. The research questions (1) What are the main themes and trends in entrepreneurial leadership research in business organizations? (2) What are the practical insights for business leaders on applying the principles of entrepreneurial leadership to achieve business organizational success? (3) What are the most commonly associated variables with EL? (4) What are the individual competencies needed to be able to effectively implement Entrepreneurial Leadership Factors in a business organization? (5) What are the outcomes arising from the implementation of EL in Business Organizations?

## Literature review

### Concept of entrepreneurial leadership in business management

New approaches to developing the necessary skills are needed in a business environment that is changing quickly so that managers can react quickly to ongoing changes ( Ercantan et al., 2024). Interestingly, the need for entrepreneurial leadership is growing every day in order to promptly adjust to continuous changes in a global economy. Entrepreneurial leadership is a distinctive style of leadership that can be present in an organization of any size, type, or age ( Renko et al., 2015). Entrepreneurial leaders perform a dual role, as entrepreneurial accelerators, they encourage followers to challenge the status quo and engage in creative problem-solving; as entrepreneurial doers, they act as role models by directly participating in entrepreneurial activities, thereby promoting vicarious learning among team members ( Iqbal et al., 2022). In business management practice, Entrepreneurial leadership plays a critical role in facilitating employee and team creativity in terms of displaying creativity-favouring behaviours that specifically fit workplace creative endeavours ( Cai et al., 2019). Entrepreneurial leaders foster an environment where innovation is not merely encouraged but embedded into daily operations through several mechanisms.

The focus on the examination of entrepreneurial leadership can traditionally be divided into two, depending on whether it is initially viewed from an entrepreneurial or leadership perspective ( Leitch & Volery, 2017). Integrating the latest technology, innovation, resources, opportunities, and added value will determine the success of a business ( Chaniago, 2023). The complexity of the relationship between organizational culture and entrepreneurial leadership and the application of a combined model of reflective practitioners and learner organizations in innovative leadership ( Shiferaw et al., 2023).

The conceptualization of EL can determine the direction of an organization and have a dynamic process ( Harrison et al., 2016). Although attention is focused, entrepreneurship and leadership are still ambiguous concepts ( Harrison et al., 2016). Shiferaw et al., (2023) conducting a systematic literature review, revealing the intricate relationships between entrepreneurial leadership, organizational learning, and organizational culture. Entrepreneurial leadership skills are still broad and largely unstructured ( Harrison et al., 2016). Norena-Chavez & Thalassinos, (2023) entrepreneurial leadership, not only at the leadership level but also throughout the organizational hierarchy. Djalil et al. (2023) demonstrating the important role of entrepreneurial leadership in improving the bank's performance through market orientation and innovation. The field of leadership and entrepreneurship provides essential insights into how individuals and organizations function and work in complex environments ( Harrison et al., 2016). Hensellek et al., (2023) entrepreneurial leadership affects performance in various kinds of organizations, such as new ventures, small- and medium-sized enterprises, and established firms.

Entrepreneurial Leadership (EL) is a leadership style that focuses on recognition, exploitation of opportunities, and empowering teams to create value in organizations. Gupta et al., (2004), added that EL includes the scenario enactment dimensions (creating a vision and mitigating uncertainty) and cast enactment (building team commitment). In an empirical context, Latif et al., (2020) shows that EL has a significant impact on the success of the project through the mediating role of the knowledge management process, such as the acquisition and utilization of strategic information. ( Mehmood, Jian, & Akram, 2020a) found that EL enhances employee creativity through knowledge sharing, with the added influence of learning orientation.

EL is also relevant in cross-cultural contexts. ( Gupta et al., 2004) identified that EL is globally recognized as a characteristic of effective leadership, although there are variations in its implementation depending on organizational culture and power hierarchies. ( Renko et al., 2015) developed the ENTRELEAD scale to quantitatively measure EL attributes, with high validity in various organizational contexts. EL requires determination to create solutions to various challenges, reducing uncertainty and risk at various stages of business development ( Hussain & Li, 2022). This leadership style is not limited to the organizational level, but rather focuses on exploiting opportunities and developing value through route clearing to achieve goals ( Hussain & Li, 2022). Although individual attention is an important component of transformational leadership, it is not a key element in EL ( Hussain & Li, 2022).

According to ( Hoang et al., 2022), EL plays an important role in improving the company's performance, while Taleb et al., (2023) emphasizes that EL helps micro, small, and medium enterprises (MSMEs) to survive and achieve success. Therefore, governments and socio-economic development institutions should focus more on developing entrepreneurial leadership skills, especially among low-income entrepreneurs ( Taleb et al., 2023). Gupta et al., (2004) explains that EL is a different type of leadership from traditional leadership styles, as it prioritizes resource mobilization and a commitment to creating value. These two challenges are interrelated, because the vision will be meaningless without supporters who are able to carry it out ( Gupta et al., 2004). Through cross-cultural research using data from Global Leadership and Organizational Behavior Effectiveness (GLOBE), EL is recognized as a universal leadership style that is relevant in various societies and is able to adapt to different social contexts ( Gupta et al., 2004).

### Theoretical foundations of EL

a) Resource-Based View (RBV)

Resource-Based View (RBV) introduced by Jay B. Barney (1991), entrepreneurial leadership (EL) is seen as a strategic resource that is able to create a competitive advantage if it meets the criteria of valuable, rare, inimitable, and non-substitutable (VRIN). In this perspective, EL is not only interpreted as a leadership style, but as an organizational capability that plays a role in identifying, developing, and orchestrating other resources. The study of Wu et al., (2021) shows that EL affects organizational performance indirectly through ambidextrous learning—a combination of exploratory and exploitative learning—which is positioned as a strategic intangible resource. The RBV perspective emphasizes that EL’s effectiveness relies heavily on its ability to build intermediary capabilities that bridge the relationship between leadership and performance.

RBV also provides a perspective that the attributes and competencies possessed by entrepreneurial leaders are a form of individual strategic resources that contribute to the performance and sustainability of the organization. The study by Al Mamun et al., (2018) shows that characteristics such as responsibility, accountability, analytical thinking, and emotional intelligence play a role as VRIN resources that affect the performance and sustainability of micro businesses. Meanwhile, Razzaque et al., (2024) found that not all EL attributes have a positive impact; Only passion and motivation consistently improve firm performance and corporate sustainable development, while innovativeness in crisis conditions can actually have a negative impact. These findings confirm that in the perspective of RBV, the strategic value of a resource is highly contextual and influenced by environmental conditions ( Al Mamun et al., 2018; Razzaque et al., 2024).

EL’s main role lies in its ability to create and strengthen organizational resources and capabilities that are drivers of competitive advantage. The effectiveness of EL in the RBV framework is more channeled through the development of strategic capabilities rather than direct influence, so that EL can be understood as a resource orchestrator that optimizes organizational resources to achieve sustainable performance and competitive advantage ( Ercantan et al., 2024; Taleb et al., 2023). EL not only has a direct impact on outcomes, but also through the process of developing, integrating, and reconfiguring knowledge-based resources that enable organizations to adapt to a dynamic environment.

b) Social Cognitive Theory (SCT)

Social Cognitive Theory (SCT) developed by Albert Bandura (1986) emphasizes that individual behavior is the result of a reciprocal interaction between personal factors (cognitive), environment, and behavior itself (triadic reciprocal causation). In the context of entrepreneurial leadership (EL), leaders play a role as an environmental determinant as well as a role model that influences the cognition and behavior of subordinates. Hussain et al., (2024) show that EL functions as a proxy agency that influences entrepreneurial intentions through two main mechanisms, namely entrepreneurial self-efficacy and identification with the leader. Through the process of vicarious learning and social encouragement, EL increases an individual’s confidence in his or her ability to be entrepreneurial, while encouraging the process of identifying values and attitudes with leaders.

Hoang et al., (2022) found that EL plays an environmental determinant that influences innovative behavior through the mediation of intrinsic motivation and trust in leader, which are personal determinants within the framework of SCT. Meanwhile, Newman, et al., (2018a) showed that EL functions as a boundary condition that strengthens the relationship between creative self-efficacy (CSE) and innovative behavior. Compared to transformational leadership and participative leadership, EL has proven to be more effective in strengthening these relationships because leaders not only inspire, but also actively act as entrepreneurial doers and entrepreneurial accelerators. This confirms that in the perspective of SCT, EL not only acts as a mediator, but also as a moderator who reinforces the influence of cognitive factors on innovative behavior.

## Methods

This literature review aims to synthesize and critically analyze existing research on the interaction between entrepreneurial leadership and organizational outcomes, focusing on research variables, hypotheses, and theoretical foundations. It offers a comprehensive overview of how different studies conceptualize and measure key variables such as innovation performance, organizational culture, and entrepreneurial success. This research employs a systematic approach, beginning with a literature search and thematic analysis. The article is structured as follows: first, defining and discussing the core characteristics of entrepreneurial leadership; second, examining various theoretical frameworks in the literature; third, reviewing common variables in the literature; and finally, analyzing prevalent themes, identifying gaps and challenges, and proposing implications for future research and practice.

Including every published article on entrepreneurial leadership was not feasible given the volume of literature retrieved; a theoretical sampling strategy following the PRISMA (Preferred Reporting Items for Systematic Reviews and Meta-Analyses) protocol was therefore adopted to identify representative and conceptually relevant studies. However, due to the extensive research in this field and the numerous articles identified through our search and screening process, including all relevant articles would be impractical and time-consuming. Therefore, a feasible alternative is employing theoretical sampling as per the PRISMA guidelines to achieve the study's objectives. Initially, articles were selected from a population of 364 articles from Scopus. To illustrate the article selection process, a PRISMA flowchart ( Helden et al., 2023) was used, comprising four main stages: identification, screening, eligibility, and inclusion. Initially, 364 articles were identified through database searches. After excluding ineligible articles (titles, abstracts, and irrelevance), 83 articles underwent full-text review, with all 83 ultimately included in the final systematic review.

This study adopts the PRISMA (Preferred Reporting Items for Systematic Reviews and Meta-Analyses) protocol to ensure a transparent and systematic literature review process. The author’s steps in conducting a PRISMA protocol consist of identifying, filtering, eligibility, and inclusion of articles that have been identified using the keywords entrepreneurial leadership. The researcher conducted an initial search on journals indexed by scopus and web of science and managed to find 364 articles with entrepreneurial leadership research topics. In the second stage, the researcher screened articles based on abstract content, obtained 221 articles that were considered relevant and discarded 137 articles because they had the same title. In the third stage, the researcher read the full articles in the background, literature review, methodology, and data availability, so that 115 articles that met the eligibility criteria were obtained. Finally, the researcher only analyzed 83 articles because they were considered to have good quality in terms of methodology and completeness of the research results presented.

PRISMA will benefit authors, editors, and peer reviewers of systematic reviews, and different users of reviews, including guideline developers, policy makers, healthcare providers, patients, and other stakeholders ( Page et al., 2021). In addition, this study incorporates bibliometric analysis to identify research trends, keyword co-occurrence, and influential publications. A bibliometric analysis is used to assess where the most active influen- tial research focus on this topic lies ( Torrijo & Giner, 2023). The bibliometric method was used in this study to obtain a database of entrepreneurial leadership. The results of the bibliometric presented the publication of articles, countries, author affiliations, and journals with graphical analysis using VOSviewer software.

**Figure 1.**
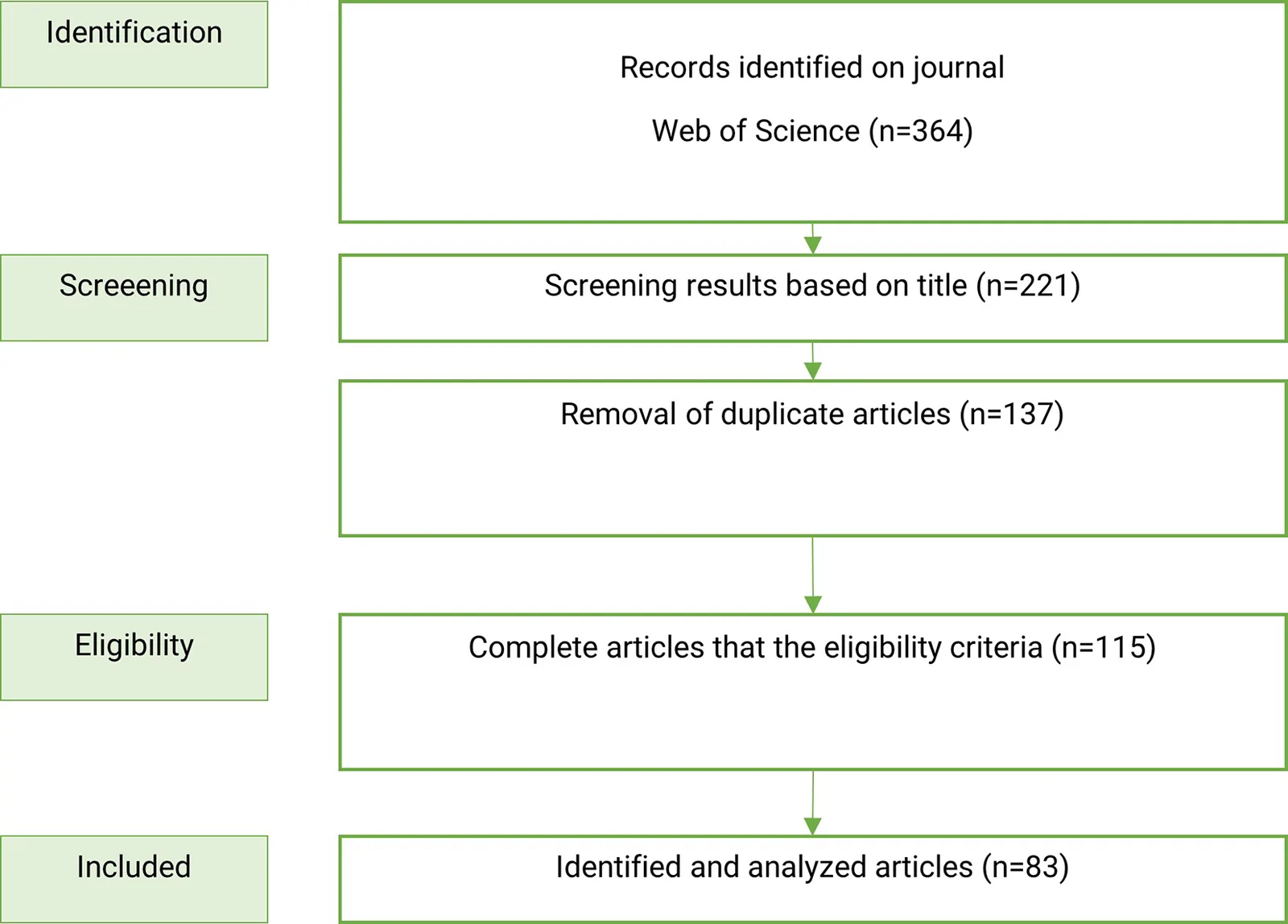
Prisma reporting.

Theoretical sampling continued until saturation and completeness were achieved, which was defined as reaching a sufficient number of research articles that identified conceptualizations pertinent to entrepreneurial leadership research. In total, 83 relevant research articles contributed to identifying and explaining these conceptualizations. Analyzing articles to elucidate entrepreneurial leadership conceptualizations (see Figure 1) necessitates constant comparison ( Helden et al., 2023). This process involves comparing codes and concepts to uncover and document their relationships, thereby refining labeled conceptualizations. These emergent conceptualizations serve as frameworks for subsequent article selection, guiding systematic deductions to select further articles. Memos are utilized to record emerging ideas and guide the selection of subsequent article samples. Each sampled article is scrutinized to discern its core contributions regarding the essence of entrepreneurial leadership or pivotal components within the conceptual framework of variables ( Helden et al., 2023) pertinent to entrepreneurial leadership research. Only after thorough analysis does the transition occur to selecting another article for examination.

## Results

**Figure 2.**
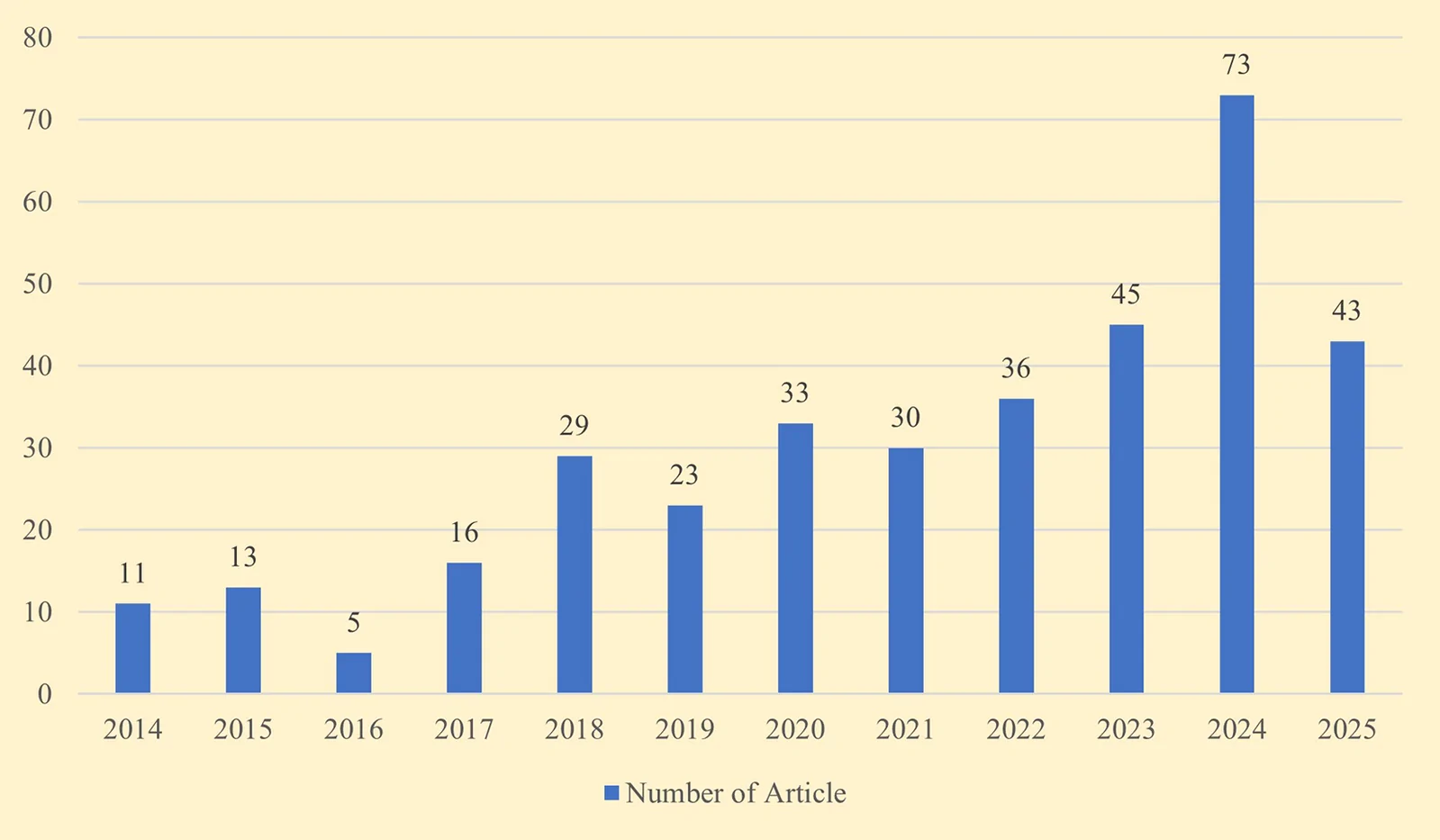
Evolution in the number of articles published.

The results bibliometric analysis starting from information in Figure 2 illustrates the trend in the number of articles published from 2014 to 2025. In 2014, 11 articles were published, and this number slightly increased to 13 in 2015. However, there was a significant drop in 2016, with only 5 articles published. Starting in 2017, the number of articles began to rise steadily, reaching 16 articles that year and then jumping to 29 in 2018. In 2019, there was a slight decrease to 23 articles, followed by an increase to 33 in 2020. In 2021, the number of publications declined slightly to 30 articles but increased again in 2022 to 36 articles, and further to 45 in 2023. The highest number of articles was recorded in 2024, with a total of 73. Although there was a slight decrease in 2025 to 43 articles, the publication volume remained significantly higher compared to the earlier years.

**Figure 3.**
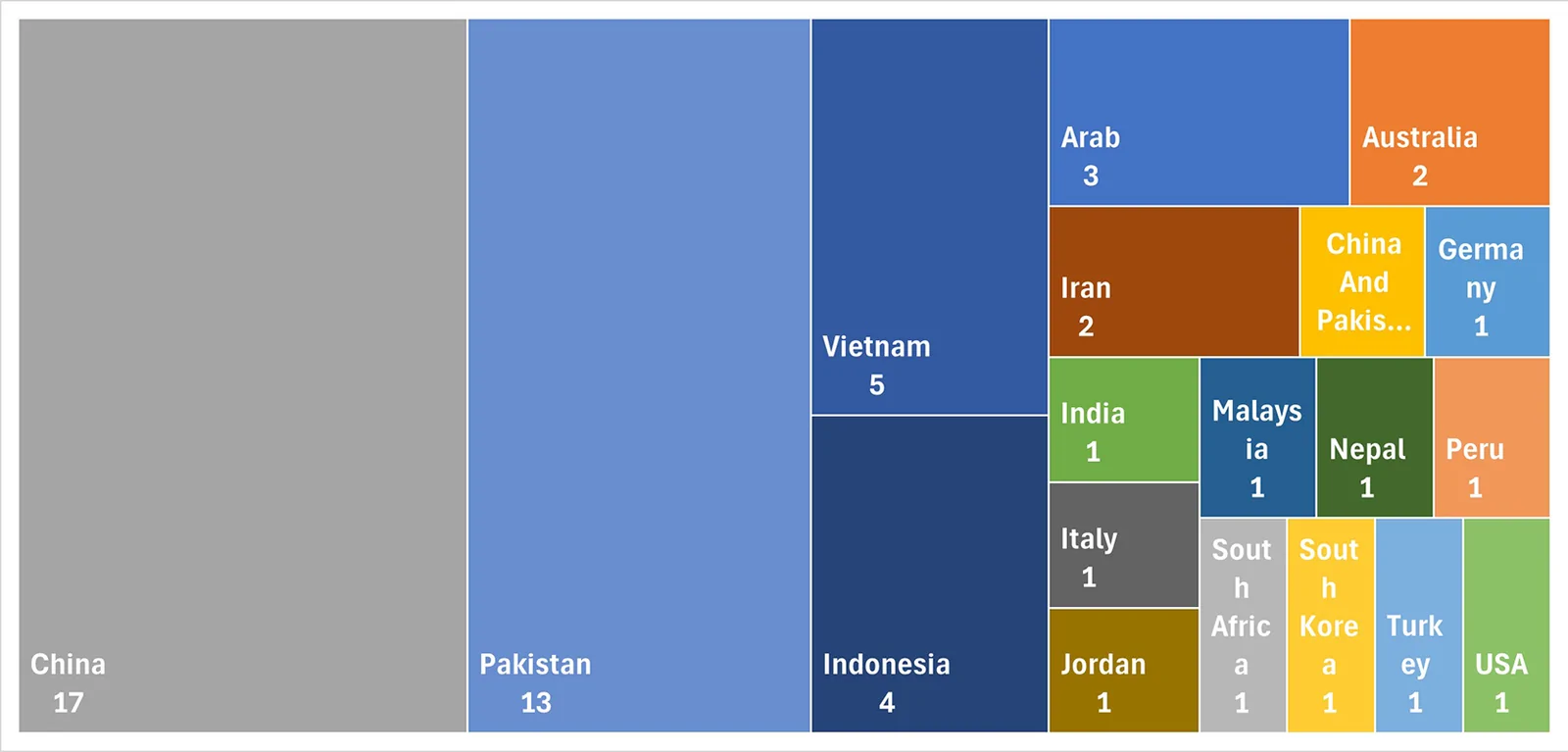
Tree map country classification.

Figure 3 is a tree map showing the classification of countries based on a specific size. In this treemap, each rectangle represents a single country, with the size of the rectangle indicating the magnitude of the value or amount associated with that country. The country of China has the largest rectangle, followed by Pakistan and Vietnam, each of which has a significant size. Indonesia also occupies a sizable area on this map. Other countries shown, albeit smaller in size, include Iran, Australia, India, South Africa, Saudi Arabia, Turkey, Italy, Nepal, USA, Jordan, Peru, Arabia, Germany, and South Korea. This treemap provides an effective visualization of the comparison between different countries in the context of their size or contribution to a given metric. This allows the reader to quickly understand the distribution and proportions between the countries involved.

Table 1 shows the ten institutions with the most relevant affiliations based on the number of publications related to entrepreneurial leadership research. The listed institutions are spread across different regions of the globe, demonstrating an international contribution to this research topic. The University of Tehran in Iran, Swinburne University of Technology in Australia, and the University of the West of Scotland in Southwest Scotland each lead with a total of 7 publications. The geographical distribution of these affiliations shows that entrepreneurial leadership research has received widespread attention from different countries and continents, with significant contributions from institutions in Iran, Australias, Scotland, China, the United Kingdom, Indonesia, and Vietnam.

**Table 1.** Top 10 most relevant affiliations.

No	Affiliation	Region	Number of publication
1	University of Tehran	Iran	7
2	Swinburne University of Technology	Australia	7
3	University of the West of Scotland	South-Western Scotland	7
4	Deakin University	Australia	6
5	University of Science and Technology Beijing	China	5
6	Nottingham Trent University	English	4
7	Universitas Padjadjaran	Indonesia	4
8	RMIT University Vietnam	Vietnam	4
9	Foreign Trade University	Vietnam	4
10	Xi'an Jiaotong University	China	4

Table 2 provides insight into the journals that cite articles related to entrepreneurial leadership research, particularly those published in journals indexed by Web of Science. Sustainability is 184 citations from 9 articles, showing significant influence in this area. The European Journal of Innovation Management, recorded 146 citations from 5 articles, while Cogent Business & Management had 37 citations from 5 articles. The Journal of Small Business Management stands out with the highest number of citations, reaching 548 citations from 4 articles, signifying its great relevance in entrepreneurial leadership research. Frontiers in Psychology and Leadership & Organization Development Journal, have 11 and 72 citations from 3 articles, respectively.

**Table 2.** Journal citation.

No	Journal	Tier	Citation	Total Articles
1	Sustainability	1	184	9
2	European Journal of Innovation Management	1	146	5
3	Cogent Business & Management	2	37	5
4	Journal of Small Business Management	1	548	4
5	Frontiers in Psychology	1	11	3
6	Leadership & Organization Development Journal	1	72	3
7	European Business Review	1	4	2
8	World Journal of Entrepreneurship, Management and Sustainable Development	1	51	2
9	Creativity And Innovation Management	1	109	2
10	Kybernetes	2	9	2
11	International Journal of Innovation Management	2	36	2
12	Journal of Business Research	1	253	2
13	Psychology Research and Behavior Management	2	55	2
14	Journal of Small Business and Enterprise Development	1	37	2
15	Journal of Service Theory and Practice	1	2	1
16	Journal of Services Marketing	1	15	1
17	Benchmarking-An International Journal	1	8	1
18	Journal of Manufacturing Technology Management	1	2	1
19	Management Decision	1	0	1

Table 3 shows the research variables that most frequently appear in studies related to entrepreneurial leadership, along with the number of studies that mention those variables and their researchers. The most dominant variable is Entrepreneurial Leadership, with 56 studies contributing, among them Awad et al., 2024a; Ercantan et al., 2024; Norena-Chavez & Romani Torres, 2024. This variable was followed by Employee Creativity and Entrepreneurial Leadership, which were mentioned in 5 studies each. Researchers such as Hou et al., 2024; Yang & Bentein, 2023 are some of the researchers who are researching Employee Creativity, while Hoang, Nguyen, et al., 2023b; Sultan et al., 2023 are researching Entrepreneurial Leadership.

**Table 3.** Research variables.

No	Variable	Total	Author
1	Entrepreneurial Leadership	61	Ahmed & Lucianetti, 2024; Ataei et al., 2024a; Awad et al., 2024; Ercantan et al., 2024; Getaneh Kebede et al., 2024; Hensellek et al., 2023; Hou et al., 2024; G. Hussain et al., 2024; Khan et al., 2024; Norena-Chavez & Romani Torres, 2024; Taleb et al., 2023; Tariq et al., 2024; Zahoor et al., 2023; Abualoush et al., 2022; Chingwena & Scheepers, 2022; Djalil et al., 2023; Hai et al., 2021; Haq & Aslam, 2023; Hoang et al., 2022, 2024; N. Hussain & Li, 2022; Pu, Ji, et al., 2022a; Yang & Bentein, 2023; Zhang et al., 2023; Alsharif et al., 2021; Bilal et al., 2021; Islam & Asad, 2024; Li et al., 2020; Mehmood, Jian, & Gilal, 2020b, Mehmood et al., 2022; Rehman et al., 2021; Wu et al., 2021; Bagheri & Harrison, 2020; Imran & Aldaas, 2020; Iqbal et al., 2022; Latif et al., 2020; Musara & Nieuwenhuizen, 2020; Wahab & Tyasari, 2020; Bagheri & Akbari, 2018; Li et al., 2020; Newman et al., 2018a; Paudel, 2019; Soomro et al., 2019; Utoyo et al., 2020; Yang et al., 2019a; Akbari et al., 2021; Fontana & Musa, 2017; Hoang et al., 2023b; Huang et al., 2014; Lin & Yi, 2023; Mehmood, Jian, & Gilal, 2020b; Sultan et al., 2023
2	Employee Creativity	5	Cai et al., 2019; Hou et al., 2024; Islam & Asad, 2024; Ximenes et al., 2019; Yang & Bentein, 2023
3	Project Success	4	Ahmed & Lucianetti, 2024; Latif et al., 2020; Norena-Chavez & Romani Torres, 2024; Tariq et al., 2024
4	Creative Self-efficacy	3	Akbari et al., 2021; Mehmood, Jian, & Akram, 2020a; Sultan et al., 2023
5	Entrepreneurial Intentions	3	G. Hussain et al., 2024; Khan, 2022; Shabeer, 2023
6	Innovative Behavior	6	Bagheri & Harrison, 2020; Ercantan et al., 2024; Hoang et al., 2022; Iqbal et al., 2022; Mehmood, Jian, & Akram, 2020a; Newman et al., 2018a
7	Intrinsic Motivation	3	Hoang et al., 2022; Sultan et al., 2023
8	Knowledge Sharing	3	Abualoush et al., 2022; Islam et al., 2024; Khawaldeh & Alzghoul, 2024
9	Team Creativity	3	Cai et al., 2019; Hoang et al., 2022; Mehmood et al., 2022
10	Affective Commitment	2	Pu, Sang, et al., 2022b; Yang et al., 2019b
11	Competitive Advantage	2	Ercantan et al., 2024; Hoang et al., 2022
12	Creative Self Efficacy	2	Cai et al., 2019; Newman et al., 2018b
13	Entrepreneurial Orientation	2	Ataei et al., 2024b; Nguyen et al., 2021
14	Entrepreneurial Self-Efficacy	2	G. Hussain et al., 2024; Yang & Bentein, 2023
15	Entrepreneurial Success	2	N. Hussain & Li, 2022; Taleb et al., 2023
16	Idea Generation	2	Bagheri & Akbari, 2018; Fontana & Musa, 2017
17	Innovation Performance	2	Fontana & Musa, 2017; Utoyo et al., 2020
18	Innovative Work Behavior	2	Abualoush et al., 2022; Li et al., 2020
19	Big Data Capability	1	Tariq et al., 2024
20	Entrepreneurial Bricolage	1	Ahmed & Lucianetti, 2024
21	Knowledge Infrastructure Capability	1	Tariq et al., 2024
22	Knowledge Management Processes	1	Latif et al., 2020
23	Knowledge Based Dynamic Capability	1	Tariq et al., 2024
24	Team Entrepreneurial Passion	1	Norena-Chavez & Romani Torres, 2024
25	Team Innovation Culture	1	Norena-Chavez & Romani Torres, 2024
26	Team Reflexivity	1	Norena-Chavez & Romani Torres, 2024

Table 4 shows the various theories that are often used in research related to entrepreneurial leadership, complete with the number of citations and the number of articles supporting the theory, as well as the names of relevant researchers. Social Cognitive Theory emerged as the most widely adopted theoretical framework among the reviewed studies, generating the highest total citation impact. The second most widely used theory is Resource Based View Theory. Social Learning Theory has 298 citations with 10 researchers. Dynamic Capability Theory was cited 35 times, Entrepreneurial Leadership Theory was cited 117 times and SDT Self-Determination Theory was cited 28 times. Four different researchers conducted research with Social Exchange Theory, Theory Of Planned Behavior, and Upper Echelons Theory. Contingency Theory, Effectuation Theory, Knowledge-Based View KBV Theory, Resource Dependence Theory, Social Identity Theory, Social Information Processing Theory are each used by 2 different articles.

**Table 4.** Report theory citation.

No	Theory	Citation	Total article	Authors
1	Social Cognitive Theory	714	19	Getaneh Kebede et al., 2024; Hoang et al., 2023b; Li et al., 2020; Rehman et al., 2021; Wahab & Tyasari, 2020; Yang & Bentein, 2023; Zahoor et al., 2023; Akbari et al., 2021; Awad et al., 2024; Bagheri & Harrison, 2020; Cai et al., 2019; Hoang et al., 2022; Malibari & Bajaba, 2022; Newman et al., 2018b; G. Hussain et al., 2024; Iqbal et al., 2022; Megheirkouni et al., 2020; Sultan et al., 2023
2	Resource Based View Theory	163	14	Abdullah Alshammari et al., 2023; Al Mamun et al., 2018; Ercantan et al., 2024; Getaneh Kebede et al., 2024; Hai et al., 2021; Khan et al., 2024b; Norena-Chavez & Romani Torres, 2024; Razzaque et al., 2024; Taleb et al., 2023; Tariq et al., 2024; Wahab & Tyasari, 2020; Wu et al., 2021
3	Social Learning Theory	298	10	Dixit et al., 2023; Iqbal et al., 2022; Islam et al., 2024; Lee et al., 2022; Mehmood, Jian, & Gilal, 2020b; Miao et al., 2019; Newman et al., 2018b; Norena-Chavez & Romani Torres, 2024; Yang & Bentein, 2023
4	Dynamic Capability Theory	35	5	Haq & Aslam, 2023; Hoang, Luu, et al., 2023a; Hoang, Nguyen, et al., 2023b; Tariq et al., 2024; Utoyo et al., 2020
5	Entrepreneurial Leadership Theory	117	5	Bagheri & Harrison, 2020; Iqbal et al., 2022; Rehman et al., 2021; Sultan et al., 2023; Yang et al., 2019b
6	Self-Determination Theory	28	5	Bilal et al., 2021, 2022; Li et al., 2020; Pu, Sang, et al., 2022b; Sipahi Dongul & Artantaş, 2023; Zhang et al., 2023
7	Social Exchange Theory	158	4	Iqbal et al., 2022; Islam & Asad, 2024; Newman et al., 2018b; Pu, Sang, et al., 2022b
8	Theory Of Planned Behavior	3	4	Dhakal et al., 2022; G. Hussain et al., 2024; Khan et al., 2024; Zhang et al., 2023
9	Upper Echelons Theory	67	4	Ali & Ghildiyal, 2023; Hensellek et al., 2023; Nor-Aishah et al., 2020; Zahoor et al., 2023
10	Contingency Theory	21	2	Shabeer, 2023; Wahab & Tyasari, 2020
11	Effectuation Theory	59	2	Ahmed & Lucianetti, 2024; Nor-Aishah et al., 2020
12	Knowledge-Based View KBV Theory	9	2	N. Hussain & Li, 2022; Tariq et al., 2024
13	Resource Dependence Theory	43	2	Hai et al., 2021; Hoang, Luu, et al., 2023a
14	Social Identity Theory	33	2	Lee et al., 2022; Musara & Nieuwenhuizen, 2020
15	Social Information Processing Theory	108	2	Iqbal et al., 2022; Miao et al., 2019
16	Absorptive Capacity Theories	30	1	Wu et al., 2021
17	Cognitive-affective Personality System Theory	8	1	Pu, Sang, et al., 2022b
18	Collective Identity Theory	32	1	Musara & Nieuwenhuizen, 2020
19	Complex Adaptive System Theory	3	1	Lin & Yi, 2023
20	Conservation Of Resources Theory	0	1	Hou et al., 2024
21	Creativity Theory	8	1	Dixit et al., 2023
22	Culturally Endorsed Leadership Theories	65	1	Miao et al., 2019
23	Empowerment Theory	8	1	Dixit et al., 2023
24	Entrepreneurial Event Theory	0	1	Shabeer, 2023
25	Entrepreneurial Vision	65	1	Miao et al., 2019
26	Entrepreneurship and Innovation Theory	29	1	Utoyo et al., 2020
27	Explorer Behavior EXPB	5	1	Rehman et al., 2021
28	Gender Theory	4	1	Megheirkouni et al., 2020
29	Institutional Theory	110	1	Yousafzai et al., 2015
30	Instrumental Stakeholder Theory	5	1	Sipahi Dongul & Artantaş, 2023
31	Intrinsic Motivation Theory	8	1	Dixit et al., 2023
32	Job Embeddedness Theory	1	1	Pu, Sang, et al., 2022b
33	Organization Learning Theory	31	1	Islam et al., 2024
34	Person-Environment Fit Theory	0	1	Shabeer, 2023
35	Self-Efficacy Theory	47	1	Li et al., 2020
36	Social Capital Theory	5	1	Sipahi Dongul & Artantaş, 2023
37	Social Construction Theory	4	1	Megheirkouni et al., 2020
38	Social Role Theory	30	1	Yang et al., 2019a
39	Strategic Entrepreneurship Theory	1	1	Zahoor et al., 2023
40	Technology Affordances and Constraints Theory	30	1	Wu et al., 2021
41	The Affective Events Theory	0	1	Hou et al., 2024
42	Theory Of Creativity	2	1	Sultan et al., 2023

In addition, the theories used in conducting EL research are Absorptive Capacity Theories, Cognitive-affective Personality System Theory, Collective Identity Theory, Complex Adaptive System Theory, Conservation Of Resources Theory, Creativity Theory, Culturally Endorsed Leadership Theories, Empowerment Theory, Entrepreneurial Event Theory, Entrepreneurial Vision, Entrepreneurship and Innovation Theory, Explorer Behavior EXPB, Gender Theory, Institutional Theory, Instrumental Stakeholder Theory, Intrinsic Motivation Theory, Job Embeddedness Theory, Organization Learning Theory, Person-Environment Fit Theory, Psychological Theories, Self-Efficacy Theory, Social Capital Theory, Social Construction Theory, Social Role Theory, Strategic Entrepreneurship Theory, Technology Affordances and Constraints Theory, The Affective Events Theory, Theory Of Creativity. Although only 1 article wrote each of these theories, there are theories that are cited many times, namely Social Information Processing Theory 108 times and Institutional Theory 110 times.

In Table 5, Resource-Based View Theory is used to base the research variables Accountability, Analytical Thinking, Competitive Advantage, Corporate Sustainable Development, Emotional Intelligence, Entrepreneurial Leadership, Entrepreneurial Leadership Skills, Entrepreneurial Opportunity Recognition, Entrepreneurial Success, Firm Performance, Innovation Capability, Innovative Behaviour, Micro-Entreprise Performance, Micro-Enterprise Sustainability, People-Oriented Leadership Style, Responsibility, Saudi Female Entrepreneurs, Task-Oriented Leadership Style ( Abdullah Alshammari et al., 2023; Al Mamun et al., 2018; Ercantan et al., 2024; Razzaque et al., 2024; Taleb et al., 2023). These resources allow companies to create strategies that are difficult for competitors to replicate ( Barney, 1991). RBV views the company as a collection of historical and “semi-permanent” resources, including physical capital, brand names, and organizational routines and capabilities. These resources are the basis for competitive advantage ( Lockett et al., 2009).

**Table 5.** Top 10 most research variables from theory.

No	Theory	Variable	Author
1	Resource-Based View Theory	a. Accountability b. Analytical Thinking c. Competitive Advantage d. Corporate Sustainable Development e. Emotional Intelligence f. Entrepreneurial Leadership g. Entrepreneurial Leadership Skills h. Entrepreneurial Opportunity Recognition i. Entrepreneurial Success j. Firm Performance k. Innovation Capability l. Innovative Behaviour m. Micro-Entreprise Performance n. Micro-Enterprise Sustainability o. People-Oriented Leadership Style p. Responsibility q. Saudi Female Entrepreneurs r. Task-Oriented Leadership Style	Abdullah Alshammari et al., 2023; Al Mamun et al., 2018; Ercantan et al., 2024; Razzaque et al., 2024; Taleb et al., 2023
2	Social Cognitive Theory SCT	a. Creative Self Efficacy b. Creative Self-efficacy c. Employee Creativity d. Employees` Innovative Behaviour e. Employees` Intellectual Agility f. Entrepreneurial Leadership g. Entrepreneurial Leadership h. Ethical Leadership i. Individual Creativity Self Efficacy j. Innovation Climate k. Innovation Work Behavior l. Innovation Work Behaviour m. Innovative Behavior n. Innovative Behaviour o. Entrepreneurial Leadership p. Intrinsic Motivation q. Proactive Work Behavior r. Service Innovative Behavior s. Support For Innovation t. Team Creative Efficacy u. Team Creativity v. Team Creativity Self-efficacy w. Trush In Leader	Awad et al., 2024; Bagheri & Harrison, 2020; Cai et al., 2019; Hoang et al., 2022; Malibari & Bajaba, 2022; Newman et al., 2018b
3	Complex Adaptive System Theory	a. Adaptive Innovation b. Boundary-Spanning Integration c. Entrepreneurial Leadership d. Exploitative Learning e. Exploratory Learning f. Resource Bricolage	Lin & Yi, 2023
4	Dynamic Capability Theory	a. Entrepreneurial Leadership b. Supply Chain Performance c. Supply Chain Resilience	Haq & Aslam, 2023
5	Knowledge-Based View KBV Theory	a. Entrepreneurial Leadership b. Entrepreneurial Success c. Knowledge Management Processes	N. Hussain & Li, 2022
6	Self-Determination Theory SDT	a. Entrepreneurial Leadership b. Entreprenurial Leadership c. Proactive Work Behavior d. Proactive Work Behavior e. Psychological Well-Being f. Sustainable Employability g. Work Uncertainty	Bilal et al., 2021, 2022
7	Social Exchange Theory SET	a. Employee Creativity b. Entrepreneurial Leadership c. Knowledge Sharing	Islam & Asad, 2024
8	Social Learning Theory	a. Employee Creative b. Entrepreneurial Leadership c. Entrepreneurial Leadership d. Team Creativity	Mehmood, Jian, & Akram, 2020a
9	Theory Of Planned Behavior	a. Entrepreneurial Leadership b. Entrepreneurial Passion c. Venture Growth Intentions	Dhakal et al., 2022
10	Upper Echelons Theory	a. Entrepreneurial Leadership b. Green Innovation c. Organizational Learning Culture d. Strategic Flexibility e. Venture Performance	Ali & Ghildiyal, 2023; Hensellek et al., 2023

**Figure 4.**
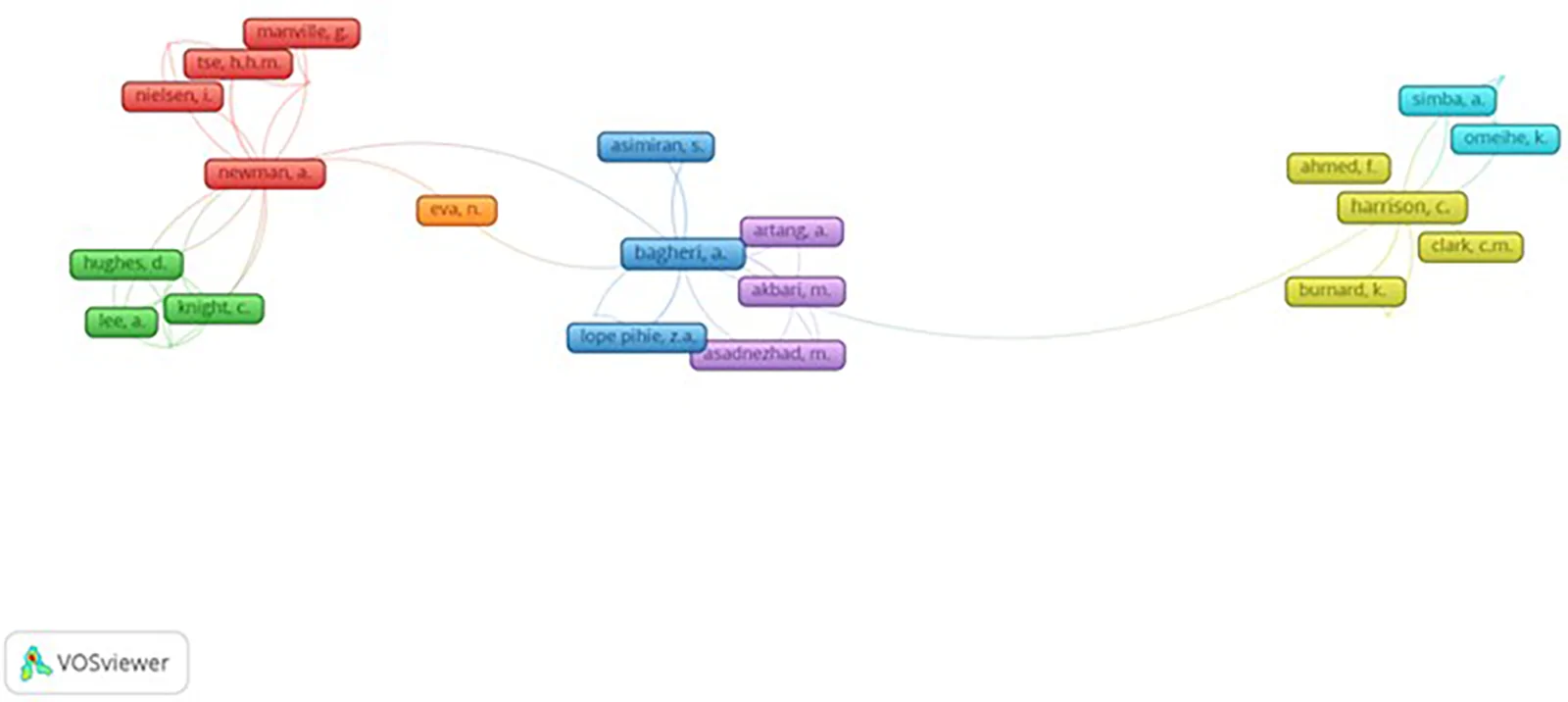
Graphical representation of authors’ network.

Graphical information from the authors' network with the theme of Entrepreneurial Leadership was created using VOSviewer in Figure 4. Each color group in this diagram shows a cluster of authors who often work together, and some authors act as liaisons between those groups. The group of authors in red has Newman, A. as its center, suggesting that this author has many collaborations with other authors in the group, such as Manville, G., Tse, H.H.M., and Nielsen, I. These writers form a close network with strong connections between them. On the other hand, the group of authors in green is made up of Hughes, D., Lee, A., and Knight, C., which also shows significant collaboration among them, albeit on a smaller scale than the red group.

The orange group of authors consists only of Eva, N., who was connected to the red group through a collaboration with Newman, A. This suggests that although Eva N. does not collaborate well within her own group, she still plays an important role in the overall network through her connection with Newman, A. Meanwhile, the group of writers in blue is led by Bagheri, A., who has many collaborations with writers such as Asimiran, S., Lope Pihie, Z.A., Artang, A., Asadnezhad, M., and Akbari, M. This group shows a strong and interconnected collaboration dynamic. On the other hand, the group of authors in yellow consists of Burnard, K., Clark, C.M., Harrison, C., and Ahmed, F., which also shows close collaboration between them. The group was connected to the cyan-colored writers' group, consisting of Simba, A. and Omeihe, K., through a collaboration involving several writers from both groups. This diagram provides a clear visual picture of how these networks of authors are interconnected and demonstrates the importance of collaboration in academic research.

Table 6 provides an overview of Entrepreneurial Leadership Characteristics influence on Business Organization Outcomes. This table shows that the variables Individual Capability, Team Behaviour, Entrepreneurial Bricolage will affect Project Success. Career Growth Opportunities have an effect on Entrepreneurial performance. Entrepreneurial Opportunity Recognition, Knowledge Management Processes, Innovation Capability have an impact on Entrepreneurial success. Employee Creativity, Intrinsic Motivation will create New product development performance. Ambidextrous Learning, Ethical Behaviour, Perceived Organizational Support, Proactiveness, Risk Taking, Social Entrepreneurial encourage the improvement of Organizational performance. Subsidiary Organizational Inertia, Subsidiary Decision Autonomy, Subsidiary Task Complexity determine Subsidiary performance. Risk taking, Entrepreneurial Competencies, Entrepreneurial intentions can improve Enterprise performance.

**Table 6.** Entrepreneurial leadership influence on employee characteristic.

Categories (Themes)	Outcomes	Exemplary papers
Individual Capability, Team Behaviour, Entrepreneurial Bricolage	Project Success	Ahmed & Lucianetti, 2024; Latif et al., 2020; Norena-Chavez & Romani Torres, 2024; Tariq et al., 2024
Career Growth Opportunities	Entrepreneurial performance	Pu, Ji, et al., 2022a
Entrepreneurial Opportunity Recognition, Knowledge Management Processes, Innovation Capability	Entrepreneurial success	Hussain & Li, 2022; Taleb et al., 2023
Employee Creativity, Intrinsic Motivation	New product development performance	Sultan et al., 2023
Ambidextrous Learning, Ethical Behaviour, Perceived Organizational Support, Proactiveness, Risk Taking, Social Entrepreneurial	Organizational performance	Imran & Aldaas, 2020; Sarmawa et al., 2020; Sipahi Dongul & Artantaş, 2023; Wu et al., 2021
Subsidiary Organizational Inertia, Subsidiary Decision Autonomy, Subsidiary Task Complexity	Subsidiary performance	Sarabi et al., 2020
Risk Taking, Entrepreneurial Competencies, Entrepreneurial intentions	Enterprise performance	Khan et al., 2024

**Figure 5.**
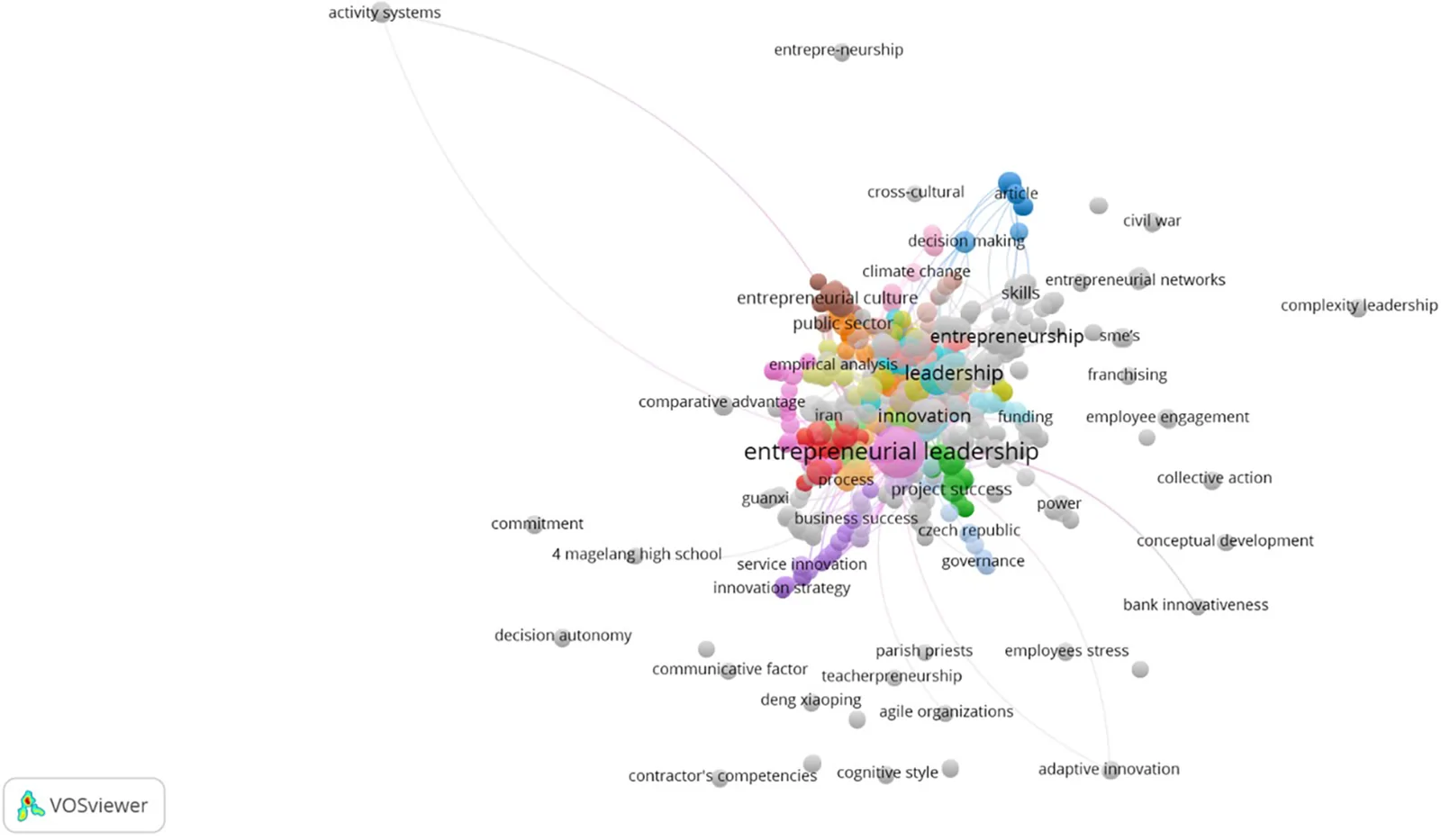
Graphical representation of co-occurrence network (frames network visualization).

Network visualization is seen in Figure 5. The most widely used keyword is entrepreneurial leadership according to the topic carried out by the author. Keywords related to individual analysis units consist of innovation, commitment, service innovation, innovation strategy, employees stress, employee enggagement, and adaptive innovation. Meanwhile, keywords related to organizational analysis units consist of business success, bank innovativeness, and project success. The leadership styles that are often related to the topic of EL.

Table 7 shows the difference between Employee Skills and Entrepreneurial Leadership Factors. To achieve sustainable competitive advantage, technical/business skills are required to achieve Big Data Analytic Capilibity, Knowledge-based Dynamic Capability, Infrastructure Capability, Dynamic Capabilities. Conceptual Skills are needed to support Product Innovation, Knowledge Management Processes, Green Innovation Behavior, Idea Generation, Strategic Flexibility, Design Thinking, Employees Intellectual Agility, and Supply Chain Resilience. Interpersonal skills focus on Innovation Work Behaviour, Employee Creativity, Proactive Work Behavior, Tacit Knowledge Sharing, Entrepreneurial Intentions, Intrinsic Motivation, and Affective Commitment. The most important skills to have are Entrepreneurial skills to create Entrepreneurial Passion, Entrepreneurial Opportunity Recognition, Individual Creativity, Self Efficacy, and Entrepreneurial Resilience.

**Table 7.** Entrepreneurial leadership factors and their exemplary papers.

Categories (Themes)	Entrepreneurial leadership factors	Exemplary papers
Technical/business skills	1. Big Data Analytic Capilibity 2. Knowledge-based Dynamic Capability 3. Knowledge 4. Infrastructure Capability 5. Dynamic Capabilities	Nguyen et al., 2021; Tariq et al., 2024
Conceptual skills	1. Product Innovation 2. Knowlegde Management Processes 3. Green Innovation Behavior 4. Idea Generation 5. Strategic Flexibility 6. Design Thinking 7. Employees` Intellectual Agility 8. Supply Chain Resilience	Ali & Ghildiyal, 2023; Bagheri & Akbari, 2018; Fontana & Musa, 2017; Haq & Aslam, 2023; Hensellek et al., 2023; Hoang, Nguyen, et al., 2023b; N. Hussain & Li, 2022; Malibari & Bajaba, 2022; Rehman et al., 2021
Interpersonal skills	1. Innovation Work Behaviour 2. Employee Creativity 3. Proactive Work Behavior 4. Tacit Knowledge Sharing 5. Entrepreneurial Intentions 6. Intrinsic Motivation 7. Affective Commitment	Abualoush et al., 2022; Awad et al., 2024; Bagheri, Akbari, et al., 2022a; Bagheri, Newman, et al., 2018b; Bilal et al., 2022; O. Ercantan & Eyupoglu, 2022; Hoang, Nguyen, et al., 2023b; G. Hussain et al., 2024; Iqbal et al., 2022; Islam et al., 2024; Khan et al., 2024; Li et al., 2020; Mehmood, Jian, & Akram, 2020a; Newman, Tse, et al., 2018a; Pu, Ji, et al., 2022a; Shabeer, 2023; Yang et al., 2019a; Zhang et al., 2023
Entrepreneurial skills	1. Entrepreneurial Passion 2. Entrepreneurial Opportunity Recognition 3. Individual Creativity Self Efficacy 4. Entrepreneurial Resilience	Dhakal et al., 2022; Khan et al., 2024; Taleb et al., 2023

Table 8 outlines the Outcomes of the Entrepreneurial Leadership Model. Individual outcomes in Entrepreneurial Leadership research consist of Big Data Analytic Capilibity, Knowledge-based Dynamic Capability, Knowledge Infrastructure Capability, Dynamic Capabilities, Product Innovation, Knowlede Management Processes, Green Innovation Behavior, Idea Generation, and Employees' Intellectual Agility. In addition, Entrepreneurial Leadership research also has an impact on team outcomes in the form of Team Creativity, Team Reflexivity, Team Innovation Culture, and Team Entrepreneurial Passion. Organizational Outcomes from Entrepreneurial Leadership research are in the form of Project Success, Entrepreneurial Performance, Entrepreneurial Success, Supply Chain Performance, Business Performance, Bank Performance, and Job Performance.

**Table 8.** Features of the entrepreneurial leadership model.

Individual outcomes	Team outcome	Organizational outcomes
1. Big Data Analytic Capilibity 2. Knowledge-based Dynamic Capability 3. Knowledge Infrastructure Capability 4. Dynamic Capabilities 5. Product Innovation 6. Knowlegde Management Processes 7. Green Innovation Behavior 8. Idea Generation 9. Employees` Intellectual Agility	1. Team Creativity 2. Team Reflexivity 3. Team Innovation Culture 4. Team Entrepreneurial Passion	1. Project Success 2. Entrepreneurial Performance 3. Entrepreneurial Success 4. Supply Chain Perfomance 5. Business Performance 6. Bank Performance 7. Job Performance

## Discussion

### Main themes of EL research

The most dominant theme was Innovative Behavior (6 articles), followed by Employee Creativity (5 articles) and Project Success (4 articles). Innovative behavior is the main focus because EL doubles as an entrepreneurial accelerator (encouraging followers to challenge the status quo) and an entrepreneurial doer (acting as a role model), thus effectively transferring innovative behaviors through observational learning mechanisms ( Iqbal et al., 2022; Newman, et al., 2018a). Employee creativity ( Cai et al., 2019; Yang & Bentein, 2023) and team creativity ( Hoang et al., 2024; Mehmood et al., 2022) is also a major concern because the characteristics of creative, visionary, and risk-taking ELs encourage employees and teams to come up with innovative solutions. Meanwhile, Creative Self-Efficacy ( Akbari et al., 2021; Sultan et al., 2023), Entrepreneurial Intentions ( Hussain et al., 2024), Intrinsic Motivation ( Hoang et al., 2024), and Knowledge Sharing ( Islam & Asad, 2024) each appeared in 3 articles as key mediation mechanisms explaining how EL affects employee behavioral outcomes and attitudes.

In addition, Project Success (4 articles) emerges as an important theme in the context of project-based industries such as IT and software development, where EL influences project success through the mediation of team capabilities and knowledge-oriented dynamic capabilities ( Norena-Chavez & Romani Torres, 2024; Tariq et al., 2024). Collectively, these eight themes show that EL’s research is dominated by behavioral outcomes (innovative behavior, creativity, knowledge sharing) with Social Cognitive Theory (SCT) and Social Learning Theory (SLT) as the main theoretical frameworks that explain the observational mechanisms of learning, role modeling, and self-efficacy. Meanwhile, the Resource-Based View (RBV) is more widely used to explain organizational level outcomes such as project success and competitive advantage through the development of knowledge-based dynamic capabilities. The dominance of innovative behavior as the most frequently researched theme confirms that EL’s main focus is to encourage innovative employee behavior as the foundation of organizational competitive advantage in an era of uncertainty.

### Theoretical trends

Social Cognitive Theory (SCT) describes how individuals shape behavior through interactions between environment, experience, and cognition. In this context, this theory is widely used to explain innovative and proactive behavior in organizations. Bilal et al., (2021) states that proactive work behavior is influenced by self-perception, past experiences, and future expectations that are in accordance with the observation and reinforcement mechanisms in the SCT. Employees with high self-confidence and social support tend to exhibit initiative and anticipatory behaviors. Bagheri et al., (2022b); Cai et al., (2019); Newman, Neesham, et al., (2018b); Sultan et al., (2023) emphasizing that an individual's belief in his or her creative self-efficacy is a central aspect of SCT. This belief encourages individuals to try new approaches, solve problems innovatively, and not be afraid to fail. Sultan et al., (2023) Show that intrinsic motivation (the internal drive to do something because it is fun or meaningful) is aligned with self-efficacy beliefs and personal values in SCT. Ercantan et al., (2024); Hoang et al., (2022); Iqbal et al., (2022) concludes that innovative behaviors are the result of a combination of self-efficacy, observational learning, and a work environment that supports experimentation. SCT describes how individuals model the behavior of innovative colleagues.

Social Cognitive Theory (SCT) emphasizes that individuals learn through observational learning, vicarious learning, and role modeling from authority figures such as leaders. In the context of EL, entrepreneurial leaders function as role models whose opportunity-oriented behaviors (proactiveness, innovativeness, risk-taking) are observed and imitated by employees ( Gupta et al., 2004; Renko et al., 2015). EL affects employee personal factors such as entrepreneurial self-efficacy ( Hussain et al., 2024; Yang & Bentein, 2023), creative self-efficacy ( Iqbal et al., 2022; Mehmood et al., 2020a), intrinsic motivation ( Hoang et al., 2022), psychological safety ( Mehmood et al., 2020b; Miao et al., 2019), and identification with the leader ( Hussain et al., 2024), which in turn drives behavioral outcomes such as innovative behavior, creativity, and entrepreneurial intentions at various levels of analysis (individual, team, organization). Cross-study findings confirm that the effectiveness of EL within the SCT framework is highly dependent on the outcomes measured, level of analysis, and organizational context ( Newman, Neesham, et al., 2018b; Norena-Chavez & Romani Torres, 2024).

Resource-Based View (RBV) ( Barney, 1991) is the second dominant theory. RBV views EL as a valuable, rare, inimitable, non-substitutable (VRIN) strategic resource that enables organizations to develop dynamic, knowledge-based capabilities to achieve sustainable competitive advantage. Based on this framework, EL acts as an enabler that facilitates the development of capabilities such as knowledge acquisition, market-sensing capability ( Hoang et al., 2024), ambidextrous learning ( Wu et al., 2021), knowledge infrastructure capability, knowledge-based dynamic capability, big data analytic capability ( Tariq et al., 2024), innovation capability ( Ercantan et al., 2024; Taleb et al., 2023), as well as entrepreneurial competencies ( Alshammari et al., 2023; Tahir et al., 2020). In contrast to the SCT approach which focuses on individual cognitive mechanisms, RBV explains how EL affects firm-level outcomes such as competitive advantage, project success, corporate sustainable development, and organisational performance through the development of organizational capabilities. The integration of these two perspectives: SCT to explain micro-mechanisms (individual cognition) and RBV to explain macro-capabilities (organizational capabilities) is necessary to comprehensively understand EL across levels of analysis.

Latif et al., (2020) argues that knowledge management processes (such as acquisition, sharing, and deployment) are core components in improving organizational innovation capabilities. KBV emphasizes that organizations must create a system that allows the effective use of knowledge. N. Hussain & Li, (2022); Taleb et al., (2023) highlighting that entrepreneurial success is heavily influenced by the way knowledge is developed and managed. KBV explained that the combination of explicit and tacit knowledge is the foundation in creating entrepreneurial value.

RBV explains that unique, unreplicable, and valuable internal resources are key to long-term competitive advantage. In this context, leadership and opportunity recognition are two forms of strategic human resources. Study by Abdullah Alshammari et al., (2023); Al Mamun et al., (2018); Ercantan et al., (2024); Getaneh Kebede et al., (2024); Hai et al., (2021); Khan et al., (2024); Norena-Chavez & Romani Torres, (2024); Razzaque et al., (2024); Taleb et al., (2023); Wahab & Tyasari, (2020); Wu et al., (2021) shows that entrepreneurial leadership is a strategic resource that affects business performance. RBV views this capability as a unique resource that allows organizations or individuals to pursue new opportunities before competitors.

### Future research opportunities

Several future research opportunities were identified from the remaining gaps in the literature. First, from a theoretical perspective, entrepreneurial leadership (EL) research is currently dominated by Social Cognitive Theory (SCT), Resource-Based View (RBV), and Social Learning Theory (SLT). However, theories such as Upper Echelons Theory, Dynamic Capability Theory, and Knowledge-Based View (KBV) are still relatively rarely used (only 4–5 articles each), even though these theories have great potential to explain how the characteristics of the top management team affect EL, how dynamic capabilities develop, and how knowledge is strategically managed. Future research is suggested to integrate EL with theories that are still underexplored such as Effectuation Theory, Institutional Theory, and Conservation of Resources Theory to enrich understanding of how entrepreneurs deal with uncertainty, how institutional contexts affect EL, as well as how psychological resources are managed in crisis situations ( Hou et al., 2024; Shabeer, 2023; Wu et al., 2021). In addition, cross-cultural research is needed because the effectiveness of EL varies depending on national cultural values such as individualism/collectivism, power distance, and Confucian values ( Lee et al., 2022; Megheirkouni et al., 2020). Research in non-business sectors such as education, healthcare, and non-profit organizations is also still very limited, even though these sectors require entrepreneurial leadership to deal with change and limited resources.

Second, from the perspective of variables and context, the mapping results show that EL research is still focused on behavioral outcomes such as innovative behavior (6 articles) and employee creativity (5 articles), while outcomes at the organizational level such as supply chain resilience, green innovation behavior, and strategic flexibility are still very rarely studied (only 1 article each). Tables 5 and 6 show that variables such as Big Data Analytic Capability, Knowledge-based Dynamic Capability, and Knowledge Infrastructure Capability are still emerging variables and require further exploration, especially in the context of digital transformation and industry 4.0 ( Tariq et al., 2024). In addition, research on EL in developing countries (except China, Pakistan, and Malaysia) is still very limited, even though different cultural and economic contexts can produce unique findings. The network visualization in Figure 5 also shows that topics such as employees stress, employee enggagement, and adaptive innovation, and adaptive innovation are still keywords with a small size (rarely used), so future research can focus on adaptation mechanisms and innovation in conditions of limited resources. Other research opportunities include testing more complex serial mediation and moderated mediation models, using longitudinal designs to test causality (as most studies are still cross-sectional), as well as comparing EL with other leadership styles such as transformational leadership, servant leadership, and digital leadership to determine EL’s unique contributions in various organizational contexts.

## Conclusion

Research on entrepreneurial Leadership has seen significant growth in the last decade, with major contributions from developed countries such as China, Pakistan, Vietnam, and Indonesia. Leading organizations such as University of Tehran. The journal “Journal of Small Business Management” emerged as the primary source for this study. Social Cognitive Theory (SCT) is the most dominant theory used in the Entrepreneurial Leadership (EL) literature, followed by Resource-Based View Theory and Social Learning Theory.

In the future, research is expected to continue to develop with a focus on digitalization and globalization in the context of entrepreneurial Leadership. The areas of research that are anticipated to be the focus in the future include the role of entrepreneurial Leadership in the context of digitalization and globalization. The research is also expected to explore more deeply the impact of new technologies and innovative business models on entrepreneurial Leadership. Knowledge management processes and innovative work behavior are identified as key mediator/moderator variables linking entrepreneurial leadership style to business performance.

The findings indicate that EL research has evolved from conceptual discussions to empirical investigations focusing on multilevel, longitudinal, and multi-theoretical integration approaches. This research shows that entrepreneurial leadership is not just an alternative leadership style, but is a strategic imperative for organizations that want to survive and thrive in an era of rapid change. The implementation of EL requires a holistic approach that includes: (1) developing individual capabilities (creative self-efficacy, entrepreneurial passion), (2) strengthening team capabilities (team reflexivity, psychological safety), (3) building organizational capabilities (innovation capability, dynamic capabilities, big data analytics). Organizations that successfully integrate EL into their daily management practices will be better prepared to face uncertainty, adapt faster to change, and ultimately achieve a sustainable competitive advantage.

This study is limited to the article selection process is based on the assessment of researchers who may contain subjectivity, although efforts have been made to minimize bias. Future research should explore cross-country comparisons and incorporate mixed-method approaches. Future research is also suggested involving two or more independent researchers in the screening and coding process, documenting inter-rater reliability, and expanding the search to additional databases such as Scopus, Emerald, and ProQuest to improve the scope and representation of the broader literature.

Research related to EL can also be done by comparing the conditions of two or more countries. EL is widely carried out in business organizations, it needs to be expanded in non-business sectors such as education and health organizations. There are still many limitations of the research conducted in this study. This research is primarily based on articles available in Scopus, which may not include all publications related to Entrepreneurial leadership, so there is a possibility of bias in the publications accessed. The EL variable can also be used as a moderation and mediation variable. This is because currently many people have conducted research using EL variables to be Variable Independent and Variable Dependent.

## Data Availability

Zenodo: Dataset: Entrepreneurial Leadership in Business Organizations: a systematic review and meta-analysis. https://doi.org/10.5281/zenodo.18425242 ( Amborowati, A., et al., 2026). This project contains the following underlying data:
1. Dataset_Entrepreneurial Leadership.xlsx 2. PRISMA flow diagram for Entrepreneurial Leadership.pdf 3.
PRISMA_Checklist_Literature Data Integration of Entrepreneurial Leadership.docx Data is available under the terms of the e Creative Commons Attribution 4.0 International license (CC-BY 4.0).
