# Supplementary material for: Entrepreneurial Leadership in Business Organizations: A Systematic Review and Meta-Analysis
Source: F1000Res. 2026 Oct 26;15:367. Originally published 2026 Mar 5. [Version 4] doi: 10.12688/f1000research.175169.4 (PMC13560040; doi:10.12688/f1000research.175169.4)
Supplement: Peer review history

# Reviewer report: Reviewer response for version 4

**DOI:** 10.5256/f1000research.212699.r533805

**Published:** 2026-10-28

**Authors:** Benny Hutahayan

**Affiliations:** 1 University of Brawijaya, Malang, East Java, Indonesia

The author has revised it well

Are the rationale for, and objectives of, the Systematic Review clearly stated?

No

Is the statistical analysis and its interpretation appropriate?

Not applicable

If this is a Living Systematic Review, is the ‘living’ method appropriate and is the search schedule clearly defined and justified? (‘Living Systematic Review’ or a variation of this term should be included in the title.)

Not applicable

Are sufficient details of the methods and analysis provided to allow replication by others?

No

Are the conclusions drawn adequately supported by the results presented in the review?

Yes

Reviewer Expertise:

Business Administration, Human Resources Management, Law, and Governance

I confirm that I have read this submission and believe that I have an appropriate level of expertise to confirm that it is of an acceptable scientific standard.

# Reviewer report: Reviewer response for version 3

**DOI:** 10.5256/f1000research.207089.r506097

**Published:** 2026-09-08

**Authors:** Diesyana Ajeng Pramesti

**Affiliations:** 1 Universitas Muhammadiyah Magelang, Magelang, Indonesia

Dear authors,

 Thank you for your revision. There are many progresses in writing to revise the manuscript.

Are the rationale for, and objectives of, the Systematic Review clearly stated?

Partly

Is the statistical analysis and its interpretation appropriate?

Partly

If this is a Living Systematic Review, is the ‘living’ method appropriate and is the search schedule clearly defined and justified? (‘Living Systematic Review’ or a variation of this term should be included in the title.)

Yes

Are sufficient details of the methods and analysis provided to allow replication by others?

Yes

Are the conclusions drawn adequately supported by the results presented in the review?

Partly

Reviewer Expertise:

Marketing Communication, Consumer Behavior, Entrepreneurial Marketing, Brand Management

I confirm that I have read this submission and believe that I have an appropriate level of expertise to confirm that it is of an acceptable scientific standard.

# Response

**Published:** 2026-09-30

**Authors:** Agustin Amborowati

Thank you very much for your positive and encouraging feedback. We sincerely appreciate the reviewer’s time, careful evaluation, and valuable comments throughout the review process. We are pleased that the revisions have improved the manuscript, and we have carefully addressed the suggestions provided to further enhance its clarity and quality.

# Reviewer report: Reviewer response for version 3

**DOI:** 10.5256/f1000research.207089.r506835

**Published:** 2026-08-06

**Authors:** Teresa Felgueira

**Affiliations:** 1 Polytechnic Institute of Guarda, Guarda, Portugal

Thank you for the opportunity to review this revised manuscript. This is my first time reviewing the paper, although it is now in its third version. Overall, the manuscript presents a comprehensive systematic review of the literature on entrepreneurial leadership in business organizations. The study synthesises recent research, identifies the main theoretical foundations, research themes, influential variables, publication trends, and proposes directions for future research. The review provides a useful overview of the evolution of entrepreneurial leadership research and offers practical implications for researchers and practitioners interested in leadership, innovation, and organisational performance.

 The manuscript has several strengths: the objectives are clearly stated, the review addresses a relevant and growing research topic, and the use of the PRISMA framework provides a transparent structure for the literature selection process. The integration of bibliometric analysis with the systematic review offers an additional perspective on publication trends, influential journals, countries, institutions, theoretical frameworks, and research variables. The manuscript is generally well organised, and the revisions made since previous versions have improved its clarity, consistency, and overall presentation.

 Regarding the review criteria:

**1. Rationale and objectives: **The rationale for conducting the review is clearly explained, and the research gap is adequately justified. The objectives and research questions are explicitly presented and provide a clear framework for the remainder of the manuscript.

**2. Methods and analysis: **The methodology is generally well described, and the authors explain the PRISMA workflow, database searches, screening process, and bibliometric procedures. However, I selected **"Partly"** because some aspects could still be described in greater detail to improve reproducibility. In particular, the manuscript would benefit from a clearer justification of the theoretical sampling approach, a more explicit explanation of how the final 83 studies were selected from the eligible articles, and a more detailed description of the quality assessment process used to determine which studies were retained. These additions would further strengthen the transparency of the review without requiring major methodological changes.

**3. Statistical analysis:** Although the title refers to a meta-analysis, the study primarily presents a systematic review supported by descriptive bibliometric analyses rather than a quantitative statistical meta-analysis. The analyses presented are appropriate for the stated objectives.

**4. Conclusions: **The conclusions are supported by the results presented throughout the manuscript. They accurately summarise the principal findings regarding publication trends, theoretical perspectives, research variables, and implications for entrepreneurial leadership research. The revised conclusion is concise and aligns well with the evidence presented.

 The manuscript is now in good condition and only a **few minor issues remain **that could further improve its quality:

1. A slightly more detailed explanation of the study selection and quality assessment process would improve methodological transparency and facilitate replication by future researchers.
2. Some tables remain very dense and could benefit from minor formatting adjustments to improve readability, although this does not affect the scientific content. The cross-referencing of figures and tables is generally appropriate. In the published PDF, some figures and tables appear separated from the corresponding discussion. As the authors already note that this results from the publisher's formatting process, no further action is required unless requested during production.

Are the rationale for, and objectives of, the Systematic Review clearly stated?

Yes

Is the statistical analysis and its interpretation appropriate?

Not applicable

If this is a Living Systematic Review, is the ‘living’ method appropriate and is the search schedule clearly defined and justified? (‘Living Systematic Review’ or a variation of this term should be included in the title.)

Not applicable

Are sufficient details of the methods and analysis provided to allow replication by others?

Partly

Are the conclusions drawn adequately supported by the results presented in the review?

Yes

Reviewer Expertise:

Entrepreneurship; Entrepreneurial Orientation;  Entrepreneurship Education; Female Entrepreneurship; Marketing; Tourism.

I confirm that I have read this submission and believe that I have an appropriate level of expertise to confirm that it is of an acceptable scientific standard.

# Response

**Published:** 2026-09-30

**Authors:** Agustin Amborowati

Thank you very much for your comprehensive and encouraging assessment of our revised manuscript. We sincerely appreciate the reviewer’s recognition of the strengths of the study and the improvements made across the previous versions. In response to the remaining suggestions, we have further revised the Methods section by providing more detailed explanations of the study selection process, including how the final 83 studies were selected from the eligible records, and by clarifying the quality assessment process used in the review. We have also reviewed the terminology and description of the analytical approach to ensure consistency with the systematic review and bibliometric analysis conducted in the study. In addition, minor formatting adjustments have been made to several dense tables to improve readability, and the cross-referencing of tables and figures has been carefully checked. We acknowledge that the positioning of some figures and tables in the published PDF may be affected by the publisher’s production process. We greatly appreciate the reviewer’s constructive comments, which have helped us further improve the transparency, clarity, and overall quality of the manuscript.

# Reviewer report: Reviewer response for version 3

**DOI:** 10.5256/f1000research.207089.r506834

**Published:** 2026-08-05

**Authors:** Benny Hutahayan

**Affiliations:** 1 University of Brawijaya, Malang, East Java, Indonesia

- The introduction is currently very limited. The problem is not elaborated in detail.
- The references used are still limited and do not sufficiently reflect developments in the literature on entrepreneurial leadership.
- It is best to avoid including too many references in a single sentence. Each reference or small group of references should be accompanied by an explanation of the findings and their relevance.
- The research gap has not been specifically formulated. The statement that the variables, principles, and contributions of entrepreneurial leadership have not been explored extensively contradicts the numerous studies already cited. The author needs to more precisely identify theoretical, methodological, contextual, or empirical gaps.
- The rationale for conducting a systematic literature review is not yet sufficiently convincing. The author needs to explain the limitations of previous studies, how this review differs from previous ones, the scope of the literature analyzed, and the new contributions it generates.
- The explanation of the four-stage review method should be moved to the Methods section. The Introduction should focus on the background, research gaps, objectives, research questions, and contributions.
- The claim that entrepreneurial leadership enhances adaptation, organizational performance, innovation, and competitive advantage must be supported by appropriate sources. A key claim should be supported by one or more truly relevant references, rather than a collection of citations that are not discussed.
- The scope of the study is inconsistent. The opening section discusses students, the public sector, individuals, and organizations, while the research questions focus on business organizations. The author must clearly define the context and unit of analysis for the study.
- The introduction should be restructured in the following order: definitions and importance of entrepreneurial leadership, research developments, limitations of previous research and reviews, research gaps, and research objectives and questions.
- The urgency and novelty of the research have not been adequately explained.
- This manuscript is a review article, but it still includes a separate section titled “Literature Review.” The author should explain the purpose of this section or consider integrating it into the introduction to ensure a more consistent manuscript structure and avoid repetition.

**Research Method**

- The PRISMA checklist needs to be reviewed and corrected to ensure that all items correspond to the actual content of the manuscript. The page numbers or sections listed in the checklist must accurately indicate the location of the requested information.
- The manuscript does not report a bias risk assessment or a methodological quality assessment of the included studies. These assessments need to be added so that readers can determine the level of credibility of the evidence underlying the synthesis. Without an assessment of bias risk and study quality, the review’s conclusions risk treating all articles as evidence with the same level of reliability. Therefore, this aspect needs to be corrected before the validity of the synthesis and the manuscript’s conclusions can be adequately assessed.

Are the rationale for, and objectives of, the Systematic Review clearly stated?

No

Is the statistical analysis and its interpretation appropriate?

Not applicable

If this is a Living Systematic Review, is the ‘living’ method appropriate and is the search schedule clearly defined and justified? (‘Living Systematic Review’ or a variation of this term should be included in the title.)

Not applicable

Are sufficient details of the methods and analysis provided to allow replication by others?

No

Are the conclusions drawn adequately supported by the results presented in the review?

Yes

Reviewer Expertise:

Business Administration, Human Resources Management, Law, and Governance

I confirm that I have read this submission and believe that I have an appropriate level of expertise to confirm that it is of an acceptable scientific standard, however I have significant reservations, as outlined above.

# Response

**Published:** 2026-10-14

**Authors:** Agustin Amborowati

Thank you for this valuable comment. We have revised the manuscript to improve the clarity, transparency, and methodological rigor of the study.

 First, in the **Introduction** section, we strengthened the research background, clarified the research gaps and rationale for conducting the systematic literature review. We have also improved the urgency and novelty of the research. We have refined each reference, including an explanation of the findings and their relevance. We have addressed the shortcomings of previous studies by highlighting the differences between our observations and those of earlier research, incorporating relevant literature, and by outlining the new contributions of this study. We have moved the four-stage research methodology from the introduction to the Methodology section. We have clearly defined the context and unit of analysis for this study. The introduction has been restructured as follows: the definition and importance of entrepreneurial leadership; the evolution of research in this field; the limitations and insights from prior studies; research gaps; the study’s objectives and research questions; and a literature review. We streamlined the manuscript structure by removing the separate **Literature Review** section, as well as incorporating it into the introduction.

 In the **Research Method** section, we reviewed and corrected the PRISMA checklist to ensure that the page numbers and section references correspond accurately to the revised manuscript. We also clarified the article selection process and the inclusion of 83 quantitative empirical studies based on the predefined eligibility criteria. In addition, we added a subsection on methodological quality and risk-of-bias assessment to explain the methodological aspects considered in evaluating the included studies.

 We sincerely appreciate the reviewer's constructive feedback, which has helped us improve the manuscript.

# Reviewer report: Reviewer response for version 2

**DOI:** 10.5256/f1000research.201017.r490471

**Published:** 2026-07-28

**Authors:** Fery Setiawan

**Affiliations:** 1 Universitas Muhammadiyah Ponorogo, Ponorogo, East Java, Indonesia

The conclusion section mentions that major contributions come from the United States and Harvard University, as well as the "Journal of Business Venturing" as the main source. However, the findings in the table show the opposite: Indonesia is the most productive country, the University of Tehran is the top affiliate, and the Journal of Small Business Management is the most cited journal. Synchronization is required in writing conclusions.

 In my opinion, the manuscript is scientifically sound, the methodology is appropriate, the results are clearly presented, and the conclusions are supported by the evidence provided. The comments I have suggested are intended only to improve the clarity and presentation of the manuscript and are not essential for its scientific validity.

Are the rationale for, and objectives of, the Systematic Review clearly stated?

Yes

Is the statistical analysis and its interpretation appropriate?

Yes

If this is a Living Systematic Review, is the ‘living’ method appropriate and is the search schedule clearly defined and justified? (‘Living Systematic Review’ or a variation of this term should be included in the title.)

Yes

Are sufficient details of the methods and analysis provided to allow replication by others?

Yes

Are the conclusions drawn adequately supported by the results presented in the review?

Yes

Reviewer Expertise:

NA

I confirm that I have read this submission and believe that I have an appropriate level of expertise to confirm that it is of an acceptable scientific standard.

# Response

**Published:** 2026-09-30

**Authors:** Agustin Amborowati

Thank you very much for the careful review and positive assessment of our manuscript. We agree with the reviewer that the previous version of the Conclusion section was not fully consistent with the findings presented in the tables.

 We have carefully rechecked the relevant results and revised the Conclusion section accordingly. Specifically, the statements referring to the United States and Harvard University as the major contributors, and the *Journal of Business Venturing* as the main source, have been corrected to accurately reflect the findings reported in the tables. The revised Conclusion now identifies Indonesia as the most productive country, the University of Tehran as the leading affiliation, and the *Journal of Small Business Management* as the most cited journal, consistent with the corresponding bibliometric results.

 This revision is intended to ensure consistency between the reported findings and their interpretation in the Conclusion section. No changes were made to the underlying methodology or analysis; the revision concerns the accurate presentation and interpretation of the results.

 We sincerely appreciate the reviewer’s positive assessment that the manuscript is scientifically sound, the methodology is appropriate, the results are clearly presented, and the conclusions are supported by the evidence. The reviewer’s comments have helped us improve the clarity, accuracy, and consistency of the manuscript.

# Reviewer report: Reviewer response for version 2

**DOI:** 10.5256/f1000research.201017.r496865

**Published:** 2026-07-20

**Authors:** Diesyana Ajeng Pramesti

**Affiliations:** 1 Universitas Muhammadiyah Magelang, Magelang, Indonesia

Thank you for the revision submitted for this manuscript. Having read version 2 in full, I must report that many of the issues raised in my first review and several very basic issues that should not occur in a manuscript prepared for an International Journal  have not been adequately resolved. Some sections appear to be more disorganized than in version. I list concrete, page referenced examples in three areas:

1. Language that has not been translated into English
2. Sentence construction that does not follow academic English conventions
3. Figure/table placement relative to where each is discussed

I have also included a mandatory revision to be completed and returned with the next revision, so that every change can be verified against a specific page and line without ambiguity.

**1. Untranslated language text**

 Example raised previously: page 15, “disitasi sebanyak 35 kali”. This is one instance of a longer passage that remains in Indonesian. Please review the whole manuscript line by line not only the passage flagged here. Since other short fragments may have been missed.

 p.14–15

*Discussion / Results, paragraph beginning “Table 4 shows the various theories...”*

 Full sentences left untranslated in Indonesian, e.g. “Social Cognitive Theory digunakan oleh 19 peneliti terdahulu dengan sitasi terbanyak yaitu 714... Dynamic Capability Theory disitasi sebanyak 35 kali, Entrepreneurial Leadership Theory disitasi sebanyak 117 kali dan Self-Determination Theory SDT disitasi sebanyak 28 kali.”

 Translate the entire passage into English and verify it reads as a coherent extension of the preceding English paragraph (currently it duplicates/restates numbers already given in Table 4, so it may be redundant rather than just untranslated).

 p.9

*Methods, paragraph beginning “Ideally, all papers on entrepreneurial leadership...”*

 Sentence structure and register are not typical of academic English (“Ideally... Alternatively...” used as informal hedging rather than a scholarly justification of sampling strategy); also “conducting a prism” (p.9, Methods) instead of “the PRISMA process/protocol”, and “entrepreneurial leadeship” (typo for “leadership”) in the same paragraph.

 Rewrite as a direct methodological justification, e.g. “Including every published article on entrepreneurial leadership was not feasible given the volume of literature retrieved; a theoretical sampling strategy following PRISMA guidelines (citation) was therefore adopted...” Correct “prism” → “PRISMA protocol” and “leadeship” → “leadership” throughout.

 p.4

*Literature review, “In business management practice...” paragraph*

 Missing space / run-on word: “in terms ofdisplaying creativity-favouring behaviours”.

 Correct to “in terms of displaying creativity-favouring behaviours”.

 p.16

*Results, Table 7 discussion paragraph*

 “Know-Source Management Processes” does not match the variable name used elsewhere (“Knowledge Management Processes”, per Table 7 and Latif et al., 2020 discussion).

 Standardize to “Knowledge Management Processes” throughout.

 p.16

*Results, network visualization paragraph*

 “Agriculture students are also quite interesting topics in EL’s research... The countries that are often used to conduct EL research is African.”  ungrammatical (subject-verb agreement) and not clearly supported by Figure 5 or the country tree map (Figure 3), which shows China/Pakistan/Vietnam/Indonesia as dominant, not African countries.

 Either remove this claim or substantiate it with a specific reference to the co-occurrence network/keyword data, and correct grammar (“the countries... are”).

**2. English academic writing style**

 Example raised: page 9, the paragraph beginning “Ideally, all papers on entrepreneurial leadership would be included in this review (Helden et al., 2023). Alternatively, a representative sample of papers relevant to conceptual development in entrepreneurial leadership could be included. However, due to the extensive research in this field...”

 This reads as informal reasoning rather than a scholarly justification of the sampling method, and it is not an isolated case similar issues (hedging phrases, run-on justification, nonstandard terminology such as ‘conducting a prism’ instead of ‘the PRISMA protocol’) recur elsewhere in the Methods section. Please have the full manuscript professionally proofread for academic English register, not only the specific sentences quoted here, since a reviewer-by-reviewer patch job is what produced the disjointed result in version 2.

**3. Figure and table placement**

 A systematic pattern across pages 4–16 figures and tables are inserted several pages before the section that actually discusses them, which forces the reader to jump back and forth between the Literature Review/Methods and the Results/Discussion. This is very likely why the manuscript now reads as more disoriented than version 1 new sub-headings were added to the Discussion (per my round-1 feedback), but the visual elements were not moved to sit near their new discussion points.

 Figure 1 (PRISMA flowchart)

 p.5

 p.9 (Methods)

 Placed 4 pages before it is explained; interrupts the Literature Review (RBV section).

 Figure 2 (Publication trend 2014–2025)

 p.5

 p.11 (Results)

 Placed 6 pages before its discussion; appears immediately under Figure 1 in the middle of the Literature Review.

 Figure 3 (Country tree map)

 p.6

 p.11 (Results)

 Placed 5 pages ahead of discussion.

 Table 1 (Top 10 affiliations)

 p.6

 p.13 (Results)

 Placed 7 pages ahead of discussion.

 Table 2 (Journal citation)

 p.7

 p.13 (Results)

 Placed 6 pages ahead of discussion.

 Table 3 (Research variables)

 p.8–9

 p.13–14 (Results)

 Placed in the middle of the Methods section, well before Results discusses it.

 Table 4 (Theory citation)

 p.10–11

 p.14–15 (Results)

 Same pattern placed several pages before discussion.

 Figure 4 (Author network)

 p.13

 p.16 (Results)

 Placed 3 pages ahead of discussion.

 Table 5 (Variables by theory)

 p.12–13

 p.16 (Results)

 Placed ahead of, and split across, unrelated text (Figure 4 discussion falls in between).

 Table 6, Figure 5, Tables 7–8

 p.14–16

 p.16–17 (Results/Discussion)

 Multiple figures/tables cluster together on p.14–16 while their explanations are spread non-sequentially across Results and Discussion.

**Recommendation:** move each figure/table to immediately follow (or closely precede, within the same subsection) the paragraph that first refers to it, consistent with standard journal layout rather than clustering all visual elements early in the manuscript.

**4. Mandatory Revision (please complete and resubmit)**

 To avoid further confusion in the next review round, please complete the table below for every comment from my first review, my previous detailed feedback, and this second feedback including the exact page and line/paragraph, where each change was made, the original text, and the revised text. An incomplete or missing log will require an additional review cycle, or you can sign in by different color in your third revision (version 3). Besides that, please succinctly describe each finding in your research that is a key point that important in the area of the topic.

**Overall Recommendation**

 I recognize the effort made in restructuring the Discussion section and moving the PRISMA description into Methods. However, given the number and nature of the remaining issues several of which are basic writing-quality matters rather than substantive methodological disagreements. I recommend **Approve with Revision**. I would ask that the next revision be checked carefully, sentence by sentence and figure by figure, before resubmission, and that the revision feedback above be returned alongside the manuscript.

Are the rationale for, and objectives of, the Systematic Review clearly stated?

Partly

Is the statistical analysis and its interpretation appropriate?

Partly

If this is a Living Systematic Review, is the ‘living’ method appropriate and is the search schedule clearly defined and justified? (‘Living Systematic Review’ or a variation of this term should be included in the title.)

Yes

Are sufficient details of the methods and analysis provided to allow replication by others?

Yes

Are the conclusions drawn adequately supported by the results presented in the review?

Partly

Reviewer Expertise:

Marketing Communication, Consumer Behavior, Entrepreneurial Marketing, Brand Management

I confirm that I have read this submission and believe that I have an appropriate level of expertise to confirm that it is of an acceptable scientific standard, however I have significant reservations, as outlined above.

# Response

**Published:** 2026-07-22

**Authors:** Agustin Amborowati

Thank you very much for your thorough reading of Version 2 and for providing detailed, constructive feedback. We sincerely appreciate the time and effort you have dedicated to helping us improve the quality and clarity of our manuscript.

 We have carefully addressed all of your concerns in this third revision (Version 3). Specifically:

1. Language and Redundancy Refined the language to adopt a more formal academic, Translate the entire passage into English, and eliminated redundant narrative recaps that duplicated data already presented in Table 4.
2. Typographical and Terminology Corrections Corrected typos and standardized terminology throughout the manuscript, including changing "terms ofdisplaying" to "terms of displaying", "conducting a prism" to " conducting a PRISMA protocol ", "entrepreneurial leadeship" to "entrepreneurial leadership", and "Know-Source Management Processes" to "Knowledge Management Processes".
3. Data Alignment Revised narrative interpretation in the Results section to ensure strict alignment with the network visualization data in Figure 5.
4. Academic Register Rewrote the Methods section to enhance the English academic writing style and provide a formal methodological rationale.
5. Figure and Table Layout Clarification Re-verified the placement of all figures and tables. In the author's submitted manuscript (Version 2 doc file), all figures and tables were correctly positioned adjacent to their respective narrative descriptions; the visual misalignment in the published PDF appears to have occurred during the publisher's automated formatting process. This layout structure has been re-asserted.
6. Conclusion Rewritten to concisely incorporate and highlight the key research findings.

To ensure maximum clarity and full transparency during this review round, we have implemented both requested methods. Every modification made in Version 2 and 3 has been highlighted in Yellow directly within the revised manuscript.

 We sincerely thank you for the feedback, which has greatly improved our manuscript. We hope the comprehensive revisions in Version 3 fully satisfy all requirements.

# Reviewer report: Reviewer response for version 1

**DOI:** 10.5256/f1000research.193130.r465504

**Published:** 2026-03-30

**Authors:** Diesyana Ajeng Pramesti

**Affiliations:** 1 Universitas Muhammadiyah Magelang, Magelang, Indonesia

My review in the article entitled “Entrepreneurial Leadership in Business Organizations: a systematic review and meta-analysis” as follows:

**Introduction**

 You stated the purpose of this SLR study to explore existing EL research, but you did not comprehensively explain the urgency of EL research and its importance to companies, as stated in the article title. It would be better to describe the role of EL in organizations, and it is implementation and then identify the research gaps that make EL research ongoing and important today. You can develop specific research questions so that the objectives of your SLR research are clearly directed. So that with the results of your SLR research, you can provide ideas for new research topics related to EL which will become challenges and research opportunities in the future.

**Literature review**

 You have explained the concept of EL in your literature review, but it seems to lack focus and is presented in general terms. You can begin by explaining what EL is conceptually based on your EL focus area. For example, EL in-Business Management. You can write about the concept of EL from various theories and previous research. You can explain the concept of EL using the Resources Based View, Upper Echelons, Dynamic Capabilities theory or transformational leadership approach that you found in your meta-analysis.

**Method**

 In Table 2. Journal citation, you already not used English in “Total artikel”. You should corrected that using English language. And then the PRISMA protocol table should be placed in the methods section, not the discussion section. Explain the PRISMA protocol in detail and the steps involved clearly. You can also explain your rationale for using the PRISMA protocol to strengthen your SLR research. For more in-depth results, you can incorporate bibliometric techniques detail into your SLR research.

**Discussion**

 In the discussion, you simply rewrite the findings of the previous SLR. For example,

 “Social Cognitive Theory (SCT) explains how individuals shape behavior through the interaction between the environment, experience, and cognition. In this context, this theory is widely used to explain innovative and proactive behavior in organizations. Bilal et al. (2021) stated that proactive work behavior is influenced by self-perception, past experiences, and future expectations, which aligns with the observation and reinforcement mechanism in SCT. SCT explains how individuals imitate the behavior of innovative colleagues. Latif et al. (2020) argue that knowledge management processes (such as acquisition, sharing, and dissemination) are core components in enhancing organizational innovation capabilities. KBV emphasizes that organizations must create systems that enable the effective use of knowledge. N. Hussain & Li (2022); Taleb et al. (2023) highlight that entrepreneurial success is strongly influenced by how knowledge is developed and managed. KBV explains that the combination of explicit and tacit knowledge is the foundation for creating entrepreneurial value.”

 Write your SLR findings in detail and clearly. It is better to create subheadings and explain each one individually based on your meta-analysis. Your SLR research results will be more interesting if you include promising EL topics that could serve as future research directions and opportunities. In discussion you can explain your uniqueness SLR finding.

**Conclusion**

 Your conclusion still be a summary of the findings you outlined earlier, such as themes, factors influencing EL, and models of entrepreneurial leadership. However, you can outline the shifts in EL research from its inception to the present day worldwide. Significant findings are interesting and more benefits that can be strategically implemented. In the conclusion, besides contributions, you should also explain the limitations of your SLR research, such as limitations in sources, etc. And the last, you should write your recommendations for further research.

Are the rationale for, and objectives of, the Systematic Review clearly stated?

Partly

Is the statistical analysis and its interpretation appropriate?

Partly

If this is a Living Systematic Review, is the ‘living’ method appropriate and is the search schedule clearly defined and justified? (‘Living Systematic Review’ or a variation of this term should be included in the title.)

Yes

Are sufficient details of the methods and analysis provided to allow replication by others?

Yes

Are the conclusions drawn adequately supported by the results presented in the review?

Partly

Reviewer Expertise:

Marketing Communication, Consumer Behavior, Entrepreneurial Marketing, Brand Management

I confirm that I have read this submission and believe that I have an appropriate level of expertise to confirm that it is of an acceptable scientific standard, however I have significant reservations, as outlined above.

# Response

**Published:** 2026-06-24

**Authors:** Agustin Amborowati

**Reviewer’s comment :**

**Introduction**

 You stated the purpose of this SLR study to explore existing EL research, but you did not comprehensively explain the urgency of EL research and its importance to companies, as stated in the article title. It would be better to describe the role of EL in organizations, and it is implementation and then identify the research gaps that make EL research ongoing and important today. You can develop specific research questions so that the objectives of your SLR research are clearly directed. So that with the results of your SLR research, you can provide ideas for new research topics related to EL which will become challenges and research opportunities in the future.

**Author’s responses:**

 The uncertain business environment makes organizations forced to continue to innovate and be able to adapt quickly to change. Entrepreneurial Leadership (EL) is able to combine opportunity recognition, innovation, and strategic decision-making in developing adaptive strategies, improving organizational performance, and being able to create a competitive advantage.

 Previous studies of EL have been focused on various theoretical perspectives, contexts, and methodologies. Understanding of variables related to EL, EL in supporting innovation, and entrepreneurial leadership principles to achieve business success has not been explored.

1. What are the main themes and trends in entrepreneurial leadership research in business organizations?
2.  What are the practical insights for business leaders on applying the principles of entrepreneurial leadership to achieve business organizational success?
3. What are the most commonly associated variables with EL?
4. What are the individual competencies needed to be able to effectively implement Entrepreneurial Leadership Factors in a business organization?
5. What are the outcomes arising from the implementation of EL in Business Organizations?

**Reviewer’s comment :**

**Literature review**

**Literature review**

 You have explained the concept of EL in your literature review, but it seems to lack focus and is presented in general terms. You can begin by explaining what EL is conceptually based on your EL focus area. For example, EL in-Business Management. You can write about the concept of EL from various theories and previous research. You can explain the concept of EL using the Resources Based View, Upper Echelons, Dynamic Capabilities theory or transformational leadership approach that you found in your meta-analysis.

**Author’s responses:**

 2.1 Concept of Entrepreneurial Leadership in Business Management

 New approaches to developing the necessary skills are needed in a business environment that is changing quickly so that managers can react quickly to ongoing changes (Ercantan et al., 2024). Interestingly, the need for entrepreneurial leadership is growing every day in order to promptly adjust to continuous changes in a global economy. Entrepreneurial leadership is a distinctive style of leadership that can be present in an organization of any size, type, or age (Renko et al., 2015). Entrepreneurial leaders perform a dual role, as entrepreneurial accelerators, they encourage followers to challenge the status quo and engage in creative problem-solving; as entrepreneurial doers, they act as role models by directly participating in entrepreneurial activities, thereby promoting vicarious learning among team members (Iqbal et al., 2021). In business management practice, Entrepreneurial leadership plays a critical role in facilitating employee and team creativity in terms ofdisplaying creativity-favouring behaviours that specifically fit workplace creative endeavours (Cai et al., 2019). Entrepreneurial leaders foster an environment where innovation is not merely encouraged but embedded into daily operations through several mechanisms.

 2.2 Theoretical Foundations of EL

 (a) Resource-Based View (RBV)

 Resource-Based View (RBV) introduced by Jay B. Barney (1991), entrepreneurial leadership (EL) is seen as a strategic resource that is able to create a competitive advantage if it meets the criteria of valuable, rare, inimitable, and non-substitutable (VRIN). In this perspective, EL is not only interpreted as a leadership style, but as an organizational capability that plays a role in identifying, developing, and orchestrating other resources. The study of Wu et al. (2021) shows that EL affects organizational performance indirectly through ambidextrous learning—a combination of exploratory and exploitative learning—which is positioned as a strategic intangible resource. The RBV perspective emphasizes that EL's effectiveness relies heavily on its ability to build intermediary capabilities that bridge the relationship between leadership and performance.

 RBV also provides a perspective that the attributes and competencies possessed by entrepreneurial leaders are a form of individual strategic resources that contribute to the performance and sustainability of the organization. The study by Al Mamun et al. (2018) shows that characteristics such as responsibility, accountability, analytical thinking, and emotional intelligence play a role as VRIN resources that affect the performance and sustainability of micro businesses. Meanwhile, Razzaque et al. (2023) found that not all EL attributes have a positive impact; Only passion and motivation consistently improve firm performance and corporate sustainable development, while innovativeness in crisis conditions can actually have a negative impact. These findings confirm that in the perspective of RBV, the strategic value of a resource is highly contextual and influenced by environmental conditions (Al Mamun et al., 2018; Razzaque et al., 2023).

 EL's main role lies in its ability to create and strengthen organizational resources and capabilities that are drivers of competitive advantage. The effectiveness of EL in the RBV framework is more channeled through the development of strategic capabilities rather than direct influence, so that EL can be understood as a resource orchestrator that optimizes organizational resources to achieve sustainable performance and competitive advantage (Taleb et al., 2023; Ercantan et al., 2024). EL not only has a direct impact on outcomes, but also through the process of developing, integrating, and reconfiguring knowledge-based resources that enable organizations to adapt to a dynamic environment.

 (b) Social Cognitive Theory (SCT)

 Social Cognitive Theory (SCT) developed by Albert Bandura (1986, 1997, 2002) emphasizes that individual behavior is the result of a reciprocal interaction between personal factors (cognitive), environment, and behavior itself (triadic reciprocal causation). In the context of entrepreneurial leadership (EL), leaders play a role as an environmental determinant as well as a role model that influences the cognition and behavior of subordinates. Hussain et al. (2024) show that EL functions as a proxy agency that influences entrepreneurial intentions through two main mechanisms, namely entrepreneurial self-efficacy and identification with the leader. Through the process of vicarious learning and social encouragement, EL increases an individual's confidence in his or her ability to be entrepreneurial, while encouraging the process of identifying values and attitudes with leaders.

 Hoang et al. (2022) found that EL plays an environmental determinant that influences innovative behavior through the mediation of intrinsic motivation and trust in leader, which are personal determinants within the framework of SCT. Meanwhile, Newman et al. (2018) showed that EL functions as a boundary condition that strengthens the relationship between creative self-efficacy (CSE) and innovative behavior. Compared to transformational leadership and participative leadership, EL has proven to be more effective in strengthening these relationships because leaders not only inspire, but also actively act as entrepreneurial doers and entrepreneurial accelerators. This confirms that in the perspective of SCT, EL not only acts as a mediator, but also as a moderator who reinforces the influence of cognitive factors on innovative behavior.

**Reviewer’s comment :**

**Method**

 In Table 2. Journal citation, you already not used English in “Total artikel”. You should corrected that using English language. And then the PRISMA protocol table should be placed in the methods section, not the discussion section. Explain the PRISMA protocol in detail and the steps involved clearly. You can also explain your rationale for using the PRISMA protocol to strengthen your SLR research. For more in-depth results, you can incorporate bibliometric techniques detail into your SLR research.

**Author’s responses:**

 “Total artikel” change to “Total Articles”

 This study adopts the PRISMA (Preferred Reporting Items for Systematic Reviews and Meta-Analyses) protocol to ensure a transparent and systematic literature review process. The author's steps in conducting a prism consist of identifying, filtering, eligibility, and inclusion of articles that have been identified using the keywords entrepreneurial leadership. The researcher conducted an initial search on journals indexed by scopus and web of science and managed to find 364 articles with entrepreneurial leadeship research topics. In the second stage, the researcher screened articles based on abstract content, obtained 221 articles that were considered relevant and discarded 137 articles because they had the same title. In the third stage, the researcher read the full articles in the background, literature review, methodology, and data availability, so that 115 articles that met the eligibility criteria were obtained. Finally, the researcher only analyzed 83 articles because they were considered to have good quality in terms of methodology and completeness of the research results presented.

 PRISMA will benefit authors, editors, and peer reviewers of systematic reviews, and different users of reviews, including guideline developers, policy makers, healthcare providers, patients, and other stakeholders (Page, 2021).

 In addition, this study incorporates bibliometric analysis to identify research trends, keyword co-occurrence, and influential publications. A bibliometric analysis is used to assess where the most active influen- tial research focus on this topic lies (Aparisi-Torrijo & Ribes-Giner, 2023). The bibliometric method was used in this study to obtain a database of entrepreneurial leadership. The results of the bibliometric presented the publication of articles, countries, author affiliations, and journals with graphical analysis using VOSviewer software.

**Reviewer’s comment :**

**Discussion**

 In the discussion, you simply rewrite the findings of the previous SLR.

 For example,

 “Social Cognitive Theory (SCT) explains how individuals shape behavior through the interaction between the environment, experience, and cognition. In this context, this theory is widely used to explain innovative and proactive behavior in organizations. Bilal et al. (2021) stated that proactive work behavior is influenced by self-perception, past experiences, and future expectations, which aligns with the observation and reinforcement mechanism in SCT. SCT explains how individuals imitate the behavior of innovative colleagues. Latif et al. (2020) argue that knowledge management processes (such as acquisition, sharing, and dissemination) are core components in enhancing organizational innovation capabilities. KBV emphasizes that organizations must create systems that enable the effective use of knowledge. N. Hussain & Li (2022); Taleb et al. (2023) highlight that entrepreneurial success is strongly influenced by how knowledge is developed and managed. KBV explains that the combination of explicit and tacit knowledge is the foundation for creating entrepreneurial value.”

 Write your SLR findings in detail and clearly.

 It is better to create subheadings and explain each one individually based on your meta-analysis.

 Your SLR research results will be more interesting if you include promising EL topics that could serve as future research directions and opportunities.

 In discussion you can explain your uniqueness SLR finding.

**Author’s responses:**

**4.1 Main Themes of EL Research**

 The most dominant theme was Innovative Behavior (6 articles), followed by Employee Creativity (5 articles) and Project Success (4 articles). Innovative behavior is the main focus because EL doubles as an entrepreneurial accelerator (encouraging followers to challenge the status quo) and an entrepreneurial doer (acting as a role model), thus effectively transferring innovative behaviors through observational learning mechanisms (Iqbal et al., 2022; Newman et al., 2018a). Employee creativity (Cai et al., 2019; Yang & Bentein, 2023) and team creativity (Hoang et al., 2022b; Mehmood et al., 2022) is also a major concern because the characteristics of creative, visionary, and risk-taking ELs encourage employees and teams to come up with innovative solutions. Meanwhile, Creative Self-Efficacy (Akbari et al., 2021; Sultan et al., 2023), Entrepreneurial Intentions (Hussain et al., 2023), Intrinsic Motivation (Hoang et al., 2022c), and Knowledge Sharing (Islam et al., 2024) each appeared in 3 articles as key mediation mechanisms explaining how EL affects employee behavioral outcomes and attitudes.

 In addition, Project Success (4 articles) emerges as an important theme in the context of project-based industries such as IT and software development, where EL influences project success through the mediation of team capabilities and knowledge-oriented dynamic capabilities (Norena-Chavez & Romani Torres, 2024; Tariq et al., 2024). Collectively, these eight themes show that EL's research is dominated by behavioral outcomes (innovative behavior, creativity, knowledge sharing) with Social Cognitive Theory (SCT) and Social Learning Theory (SLT) as the main theoretical frameworks that explain the observational mechanisms of learning, role modeling, and self-efficacy. Meanwhile, the Resource-Based View (RBV) is more widely used to explain organizational level outcomes such as project success and competitive advantage through the development of knowledge-based dynamic capabilities. The dominance of innovative behavior as the most frequently researched theme confirms that EL's main focus is to encourage innovative employee behavior as the foundation of organizational competitive advantage in an era of uncertainty.

**4.2 Theoretical Trends**

 Social Cognitive Theory (SCT) (Bandura, 1986) emerged as the most dominant theoretical framework in entrepreneurial leadership (EL) research. This theory emphasizes that individuals learn through observational learning, vicarious learning, and role modeling from authority figures such as leaders. In the context of EL, entrepreneurial leaders function as role models whose opportunity-oriented behaviors (proactiveness, innovativeness, risk-taking) are observed and imitated by employees (Gupta et al., 2004; Renko et al., 2015). EL affects employee personal factors such as entrepreneurial self-efficacy (Hussain et al., 2024; Yang & Bentein, 2023), creative self-efficacy (Iqbal et al., 2021; Mehmood et al., 2020), intrinsic motivation (Hoang et al., 2022), psychological safety (Miao et al., 2018; Mehmood et al., 2021), and identification with the leader (Hussain et al., 2024), which in turn drives behavioral outcomes such as innovative behavior, creativity, and entrepreneurial intentions at various levels of analysis (individual, team, organization). Cross-study findings confirm that the effectiveness of EL within the SCT framework is highly dependent on the outcomes measured, level of analysis, and organizational context (Newman et al., 2017; Norena-Chavez & Romani Torres, 2024).

 Resource-Based View (RBV) (Barney, 1991) is the second dominant theory. RBV views EL as a valuable, rare, inimitable, non-substitutable (VRIN) strategic resource that enables organizations to develop dynamic, knowledge-based capabilities to achieve sustainable competitive advantage. Based on this framework, EL acts as an enabler that facilitates the development of capabilities such as knowledge acquisition, market-sensing capability (Hoang et al., 2024), ambidextrous learning (Wu et al., 2021), knowledge infrastructure capability, knowledge-based dynamic capability, big data analytic capability (Tariq et al., 2023), innovation capability (Taleb et al., 2023; Ercantan et al., 2024), as well as entrepreneurial competencies (Alshammari et al., 2023; Razzaque et al., 2023). In contrast to the SCT approach which focuses on individual cognitive mechanisms, RBV explains how EL affects firm-level outcomes such as competitive advantage, project success, corporate sustainable development, and organisational performance through the development of organizational capabilities. The integration of these two perspectives: SCT to explain micro-mechanisms (individual cognition) and RBV to explain macro-capabilities (organizational capabilities) is necessary to comprehensively understand EL across levels of analysis.

**4.3 Future Research Opportunities (WAJIB ADA)**

 Several future research opportunities were identified from the remaining gaps in the literature. First, from a theoretical perspective, entrepreneurial leadership (EL) research is currently dominated by Social Cognitive Theory (SCT), Resource-Based View (RBV), and Social Learning Theory (SLT). However, theories such as Upper Echelons Theory, Dynamic Capability Theory, and Knowledge-Based View (KBV) are still relatively rarely used (only 4-5 articles each), even though these theories have great potential to explain how the characteristics of the top management team affect EL, how dynamic capabilities develop, and how knowledge is strategically managed. Future research is suggested to integrate EL with theories that are still underexplored such as Effectuation Theory, Institutional Theory, and Conservation of Resources Theory to enrich understanding of how entrepreneurs deal with uncertainty, how institutional contexts affect EL, as well as how psychological resources are managed in crisis situations (Hou et al., 2024b; Shabeer, 2023; Wu et al., 2021). In addition, cross-cultural research is needed because the effectiveness of EL varies depending on national cultural values such as individualism/collectivism, power distance, and Confucian values (Lee et al., 2022; Meghierkouni et al., 2019). Research in non-business sectors such as education, healthcare, and non-profit organizations is also still very limited, even though these sectors require entrepreneurial leadership to deal with change and limited resources.

 Second, from the perspective of variables and context, the mapping results show that EL research is still focused on behavioral outcomes such as innovative behavior (6 articles) and employee creativity (5 articles), while outcomes at the organizational level such as supply chain resilience, green innovation behavior, and strategic flexibility are still very rarely studied (only 1 article each). Tables 5 and 6 show that variables such as Big Data Analytic Capability, Knowledge-based Dynamic Capability, and Knowledge Infrastructure Capability are still emerging variables and require further exploration, especially in the context of digital transformation and industry 4.0 (Tariq et al., 2024). In addition, research on EL in developing countries (except China, Pakistan, and Malaysia) is still very limited, even though different cultural and economic contexts can produce unique findings. The network visualization in Figure 5 also shows that topics such as absorptive capability, entrepreneurial bricolage, and adaptive innovation are still keywords with a small size (rarely used), so future research can focus on adaptation mechanisms and innovation in conditions of limited resources. Other research opportunities include testing more complex serial mediation and moderated mediation models, using longitudinal designs to test causality (as most studies are still cross-sectional), as well as comparing EL with other leadership styles such as transformational leadership, servant leadership, and digital leadership to determine EL's unique contributions in various organizational contexts.

**Reviewer’s comment :**

**Conclusion**

 Your conclusion still be a summary of the findings you outlined earlier, such as themes, factors influencing EL, and models of entrepreneurial leadership.

 However, you can outline the shifts in EL research from its inception to the present day worldwide.

 Significant findings are interesting and more benefits that can be strategically implemented.

 In the conclusion, besides contributions, you should also explain the limitations of your SLR research, such as limitations in sources, etc. And the last, you should write your recommendations for further research.

**Author’s responses:**

 The findings indicate that EL research has evolved from conceptual discussions to empirical investigations focusing on multilevel, longitudinal, and multi-theoretical integration approaches. This research shows that entrepreneurial leadership is not just an alternative leadership style, but is a strategic imperative for organizations that want to survive and thrive in an era of rapid change. The implementation of EL requires a holistic approach that includes: (1) developing individual capabilities (creative self-efficacy, entrepreneurial passion), (2) strengthening team capabilities (team reflexivity, psychological safety), (3) building organizational capabilities (innovation capability, dynamic capabilities, big data analytics). Organizations that successfully integrate EL into their daily management practices will be better prepared to face uncertainty, adapt faster to change, and ultimately achieve a sustainable competitive advantage.

 This study is limited to *the article selection process is based on the assessment of researchers who may contain subjectivity, although efforts have been made to minimize bias. *Future research should explore cross-country comparisons and incorporate mixed-method approaches. Future research is also suggested involving two or more independent researchers in the screening and coding process, documenting inter-rater reliability, and expanding the search to additional databases such as Scopus, Emerald, and ProQuest to improve the scope and representation of the broader literature.
